# Supplementary material for: Mislocalization of pathogenic RBM20 variants in dilated cardiomyopathy is caused by loss-of-interaction with Transportin-3
Source: Nat Commun. 2023 Jul 18;14:4312. doi: 10.1038/s41467-023-39965-6 (PMC10353998; doi:10.1038/s41467-023-39965-6)
Supplement: Supplementary file 1 — Supplementary Information [file 41467_2023_39965_MOESM1_ESM.pdf]

Supplementary Materials for

# Mislocalization of pathogenic RBM20 variants in dilated cardiomyopathy is caused by loss-of-interaction with Transportin-3

Julia Kornienko, Marta Rodríguez-Martínez, Kai Fenzl, Florian Hinze, Daniel Schraivogel, Markus Grosch, Brigit Tunaj, Dominik Lindenhofer, Laura Schraft, Moritz Kueblbeck, Eric Smith, Chad Mao, Emily Brown, Anjali Owens, Ardan M. Saguner, Benjamin Meder, Victoria Parikh, Michael Gotthardt, and Lars M. Steinmetz *

* - corresponding author, electronic address: larsms@stanford.edu

This PDF file includes:
Supplementary Figures 1-11
Legends for supplementary figures 1-11
Supplementary Tables 1-5
References

Other Supplementary Materials for this manuscript include the following:
Supplementary data 1-14
Source data

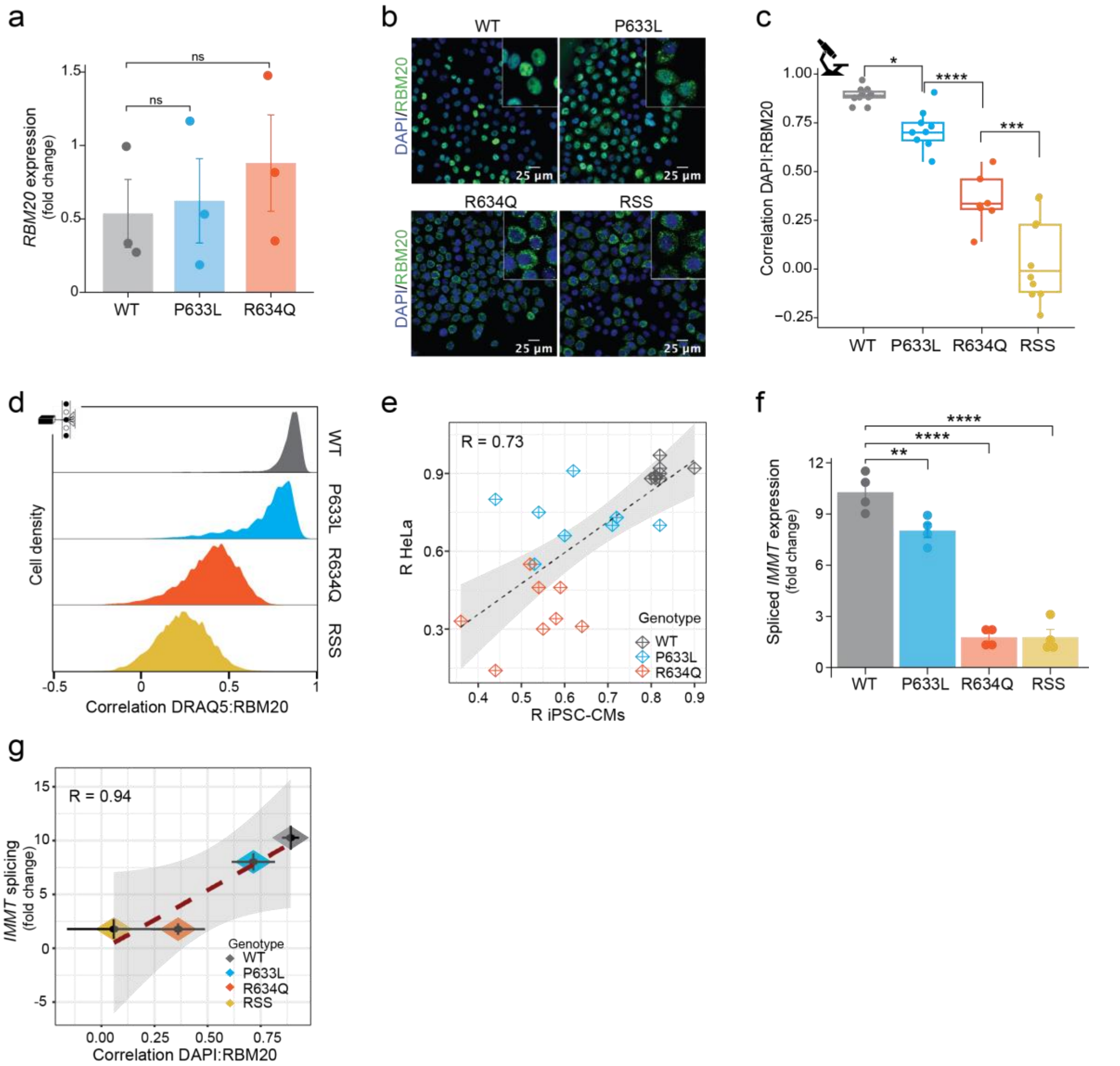


**Supplementary Figure 1. *RBM20* expression in iPSC-CMs and modelling RBM20 localization in HeLa reporter lines.** (**a**) qPCR analysis of *RBM20* expression in RBM20-WT, -P633L, or -R634Q iPSC-CMs, three biological replicates. Data is normalized to *GAPDH* expression and displayed as fold change versus the first replicate of the WT line, means with standard errors indicated. (**b**) Confocal microscopy images of HeLa eGFP-WT, -P633L, -R634Q, or -R634Q-S635E-S637E(RSS)-RBM20 reporter lines, n=3. RSS was used as the most severely mislocalized control with three out of six residues in the PRSRSP mutation hotspot stretch being substituted. (**c**) Quantification of RBM20 co-localization with DAPI based on the confocal microscopy data shown in panel (b). Each dot represents a Pearson R-value for at least ten cells, data are combined from three biological replicates. Boxplots display quartiles Q1, Q2 (center), and Q3, with whiskers extending to the furthest data point that is within 1.5 times the IQR. (**d**) ICS-based quantification of DRAQ5:RBM20 correlation. (**e**) Pearson correlation coefficient R values for DAPI:RBM20 correlation in iPSC-CMs (x axis) and HeLa (y axis) are presented as dots, each dot stands for at least five cells analyzed. Linear regression between the two is displayed as a dashed line, and regression standard error is displayed in gray. (**f**) qPCR analysis of *IMMT* exon 6 splicing in the HeLa reporter lines. Data is normalized to *GAPDH* expression and displayed as fold change versus the parental line, from two biological replicates, with two technical replicates for each of them, means with standard errors indicated. (**g**). Means of *IMMT* splicing fold change (data from panel (f), y axis) and means of Pearson correlation coefficient R for DAPI:RBM20 correlation (data from panel (c), x axis). Linear regression between the two is displayed as a dashed line, and regression standard error is displayed in grey. Standard errors are indicated as black solid lines. Ns = not significant, * - $p < 0.05$, ** - $p < 0.01$, *** - $p < 0.001$, **** - $p < 0.0001$ one-way ANOVA with Tukey's HSD post-test, two-tailored.

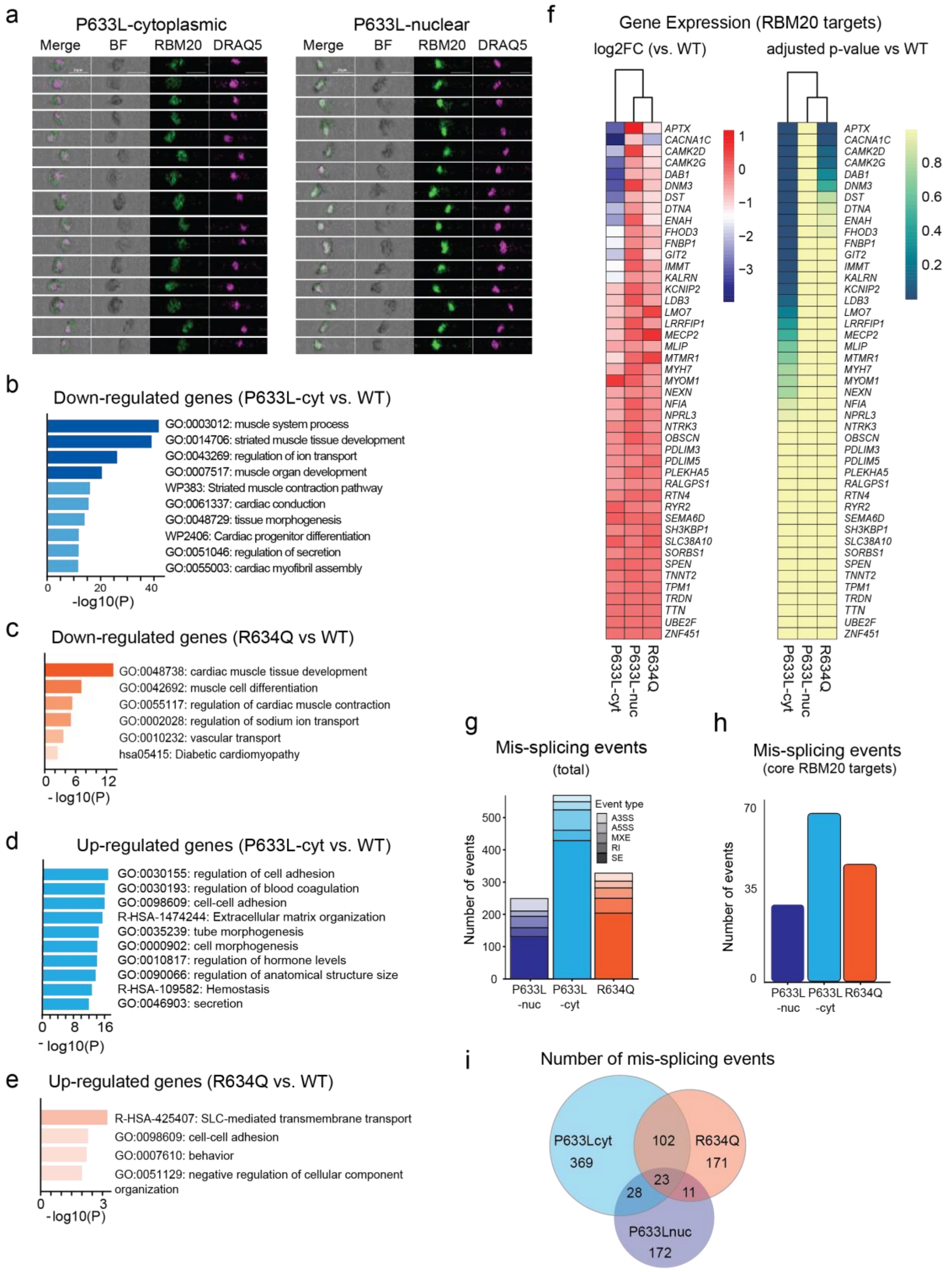


**Supplementary Figure 2. ICS-based isolation of P633L-nuc and P633L-cyt iPSC-CMs, RNA-sequencing analysis.** (**a**) Representative ICS images of P633L-iPSC-CMs in the respective sorting gates. (**b**) Pathway enrichment analysis for genes significantly down-regulated in P633L-cyt versus WT. (**c**) Pathway enrichment analysis for significantly down-regulated genes in RBM20-R634Q versus -WT iPSC-CMs. (**d**) Pathway enrichment analysis for significantly up-regulated genes in RBM20-P633L-cyt versus WT iPSC-CMs. (**e**) Pathway enrichment analysis for significantly up-regulated genes in RBM20-R634Q versus -WT iPSC-CMs.

Log10(P) values for pathway enrichment analyses were calculated by Metascape[1] for panels b-e. (**f**) Gene expression log2 fold changes and adjusted p-values (Wald test with Benjamini-Hochberg correction, calculated by DeSeq2[2]) for the core RBM20 targets, compared to the RBM20-WT iPSC-CMs. (**g**) Total number of genome-wide differential splicing events (SE = exon skipping, RI = intron retention, MXE = mutually exclusive exons, A5SS = alternative 5' starting site, A3SS = alternative 3' starting site, FDR < 0.01, inclusion level difference > 0.05, RMATS[3]) compared to the RBM20-WT iPSC-CMs. (**h**) Total number of differential splicing events (FDR < 0.01, inclusion level difference > 0.05, RMATS) per core RBM20 target gene, compared to the RBM20-WT iPSC-CMs. (**i**). Numbers and relative overlaps of all significant AS events from panel (g) in pairwise comparisons to RBM20-WT iPSC-CMs. Three biological replicates were used for all analyses.

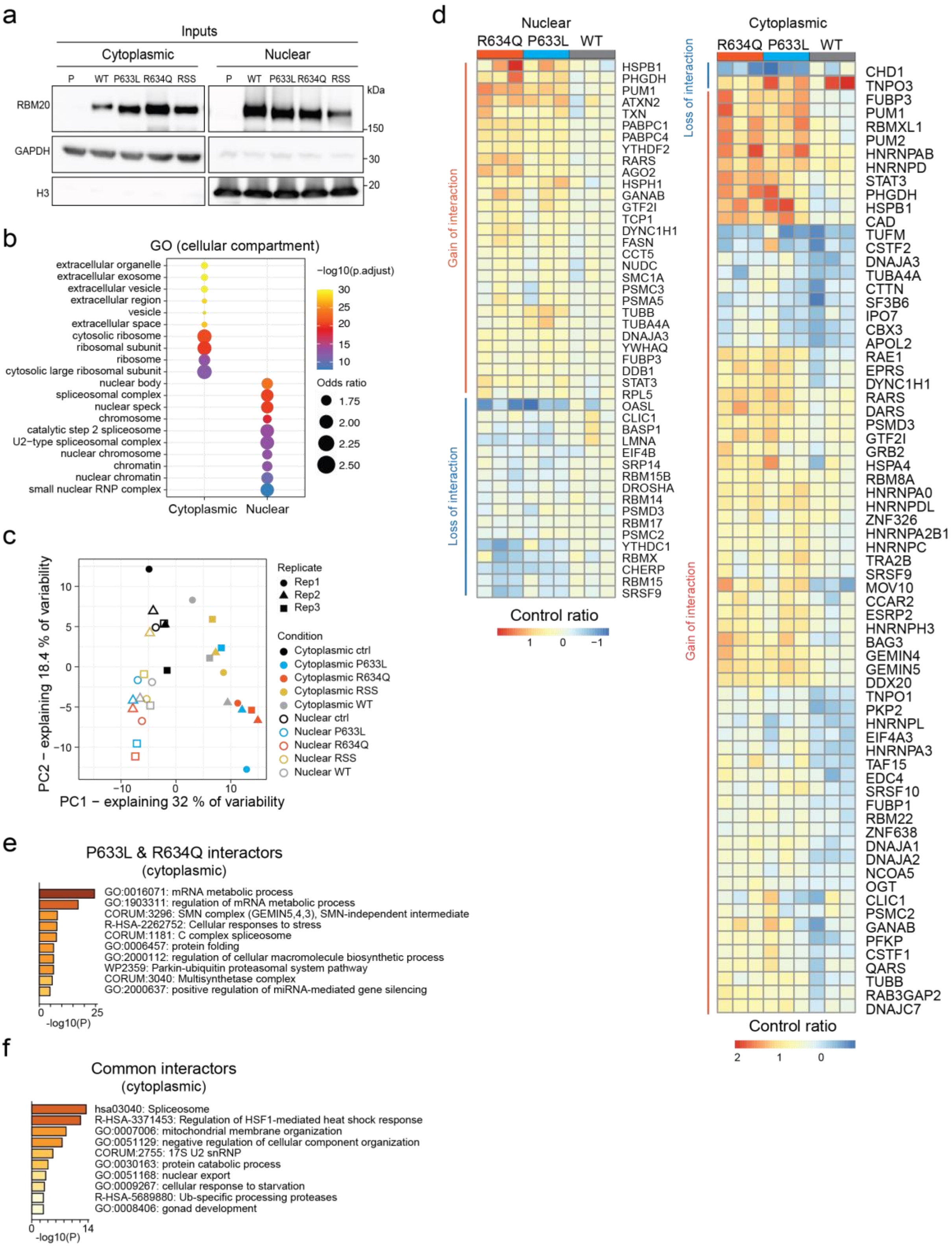

**Supplementary Figure 3. Mass spectrometry analysis of RBM20 interactors in cellular fractions of HeLa reporter lines.** (**a**) Western blot analysis of RBM20, GAPDH, and histone H3 levels in the input samples for mass spectrometry after cellular fractionation (P = parental HeLa line used as a no bait control), n=3. (**b**) GO analysis of proteins identified by mass spectrometry in the input fractions (clusterProfiler v.4.4.4). (**c**) PCA analysis for all detected by mass spectrometry peptides co-immunoprecipitating with eGFP-RBM20, ratios versus the parental line (labelled as ctrl). (**d**) Detected differential interactors between WT and mutant RBM20 in the nuclear or

cytoplasmic fractions (corresponding to Fig. 1l). Control ratio represents a ratio to the negative no bait control. Three biological replicates are shown. (**e**) Pathway enrichment analysis for proteins common between RBM20-P633L and -R634Q that are not present in the RBM20-WT; cytoplasmic fractions. **(f)** Pathway enrichment analysis for proteins common between the RBM20-WT, -P633L, and -R634Q in the cytoplasmic fraction. Log10(P) values for pathway enrichment analyses were calculated by Metascape[1] for panels (e),(f).

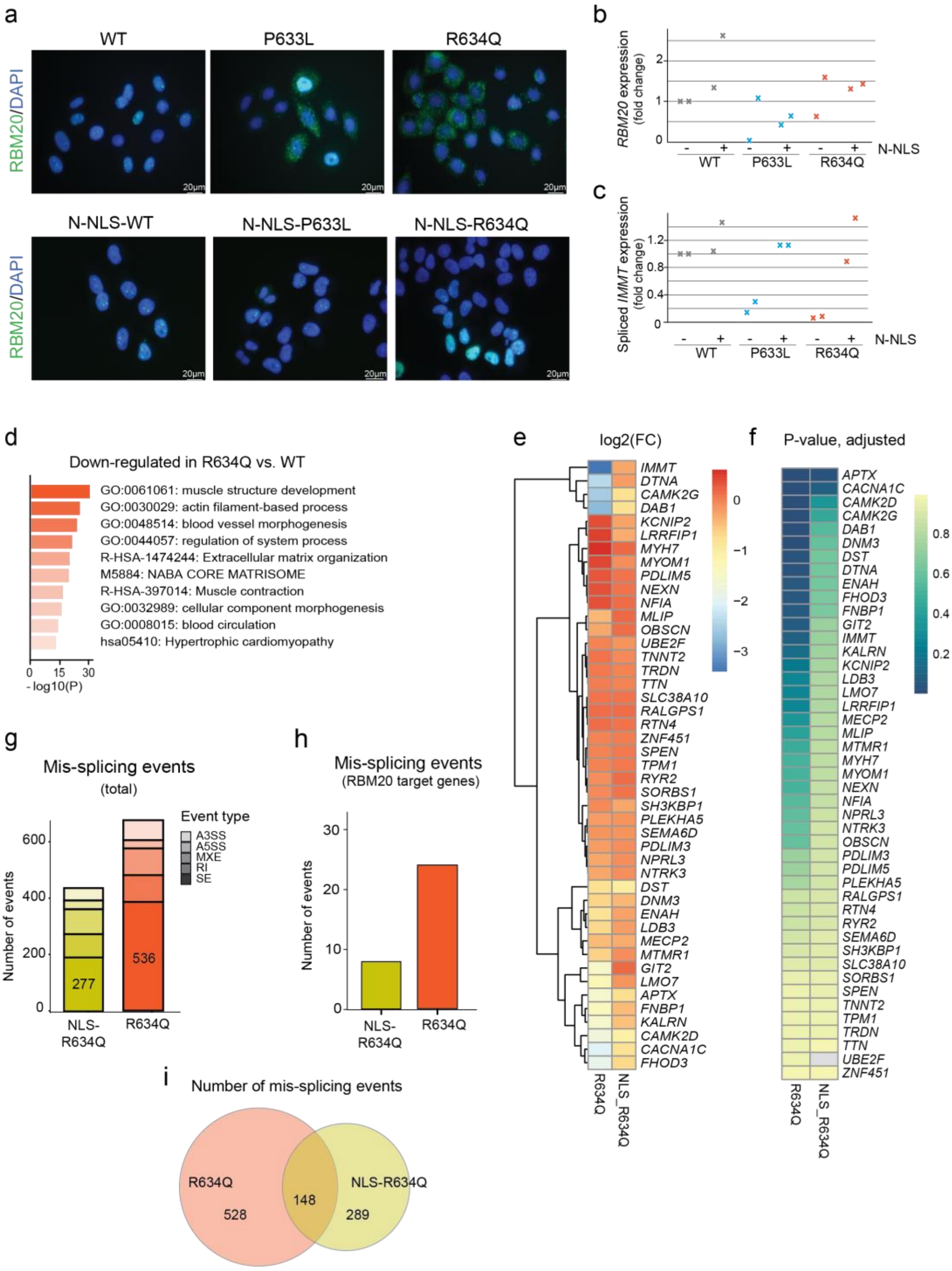


**Supplementary Figure 4. NLS-tagging of RBM20 variants rescues splicing.** (**a**) Microscopy analysis of eGFP-RBM20 localization in HeLa cells stably expressing the indicated constructs, n=3. (**b**) qPCR analysis of *RBM20* expression and (**c**) *IMMT* exon 6 splicing in HeLa cells. Data are normalized to *GAPDH* expression and displayed as mean fold change versus the eGFP-WT-RBM20 line with indicated standard errors, two biological replicates. (**d**) Pathway enrichment analysis for genes significantly down-regulated in eGFP-R634Q compared to eGFP-WT. Log10(P) values for pathway enrichment analyses were calculated by Metascape[1] (**e**) Gene expression log2 fold changes and (**f**) adjusted p-values (Wald test with Benjamini-Hochberg correction, calculated by DeSeq2[2]) for the core RBM20 target genes compared to the RS635FS iPSC-CMs expressing eGFP-WT-RBM20. (g) Total number of genome-wide differential splicing events (SE = exon skipping, RI = intron retention, MXE = mutually exclusive exons, A5SS = alternative 5’ starting site, A3SS = alternative 3’ starting site, FDR < 0.01, inclusion level

difference > 0.1, rMATS[3]) compared to the RBM20-WT iPSC-CMs. (h) Total number of differential splicing events (FDR < 0.01, inclusion level difference > 0.1, rMATS[3]) per core RBM20 target gene, compared to the RBM20-WT iPSC-CMs. (i) Numbers and relative overlaps of all significant AS events from panel (g) in pairwise comparisons to RBM20-WT iPSC-CMs. Three biological replicates were used for the analyses.

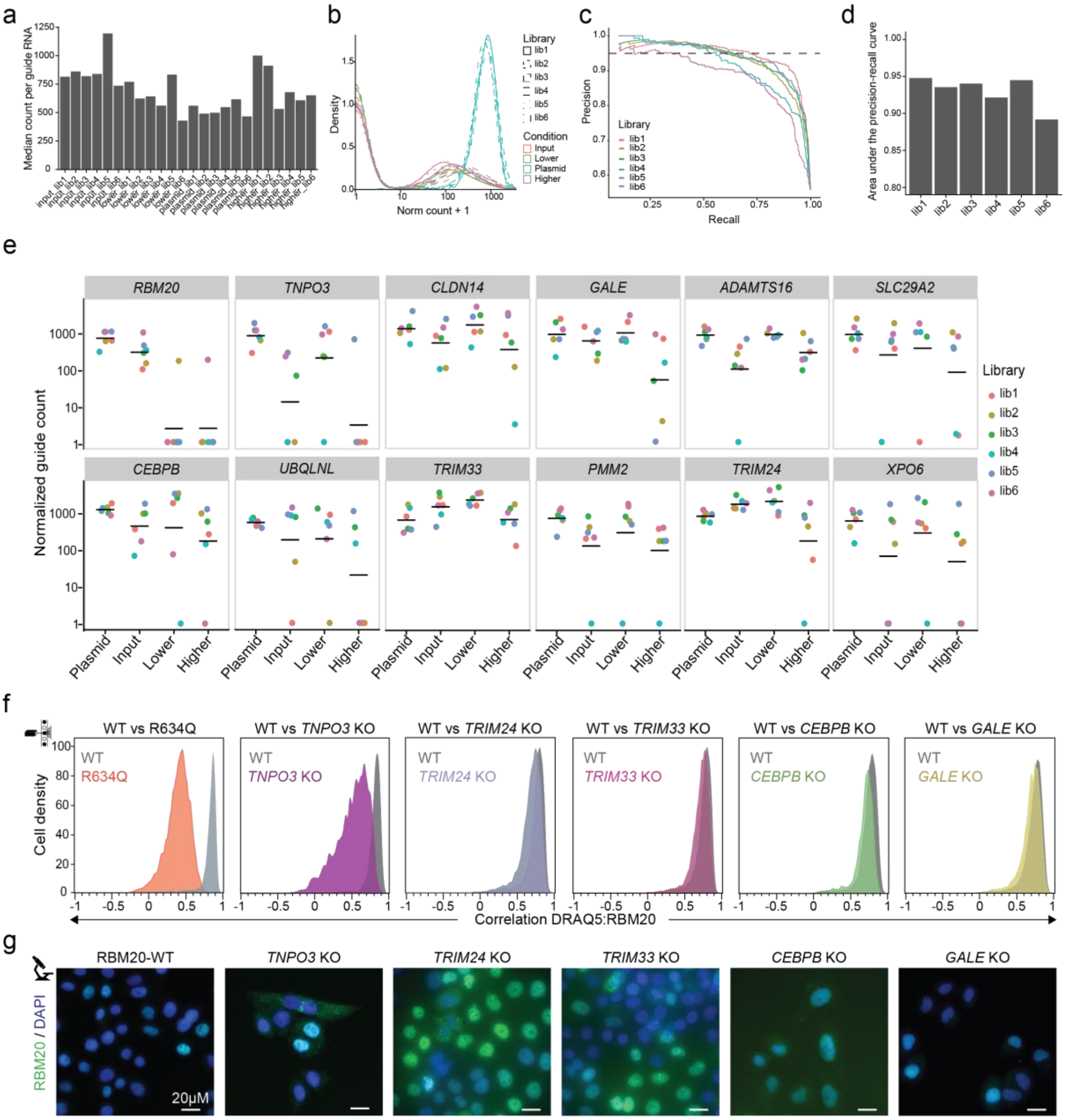

**Supplementary Figure 5. Quality control characteristics of the genome-wide CRISPR-screen, single knockouts testing for the selected hits.** (**a**). Median read count per gRNA for the six genome-wide libraries[4] per sample. (**b**) The distributions of normalized counts per library show abundance changes of gRNAs targeting core-essential genes in the unsorted and sorted samples compared to the plasmid library. Core-essential and non-essential gene annotations are based on[5]. (**c**) Precision-recall-curves indicate how well reference core essential and non-essential genes can be separated based on gRNA abundance changes in the unsorted samples after Cas9 induction compared to the plasmid library. (**d**) Areas under the precision recall curves shown in panel (c). (**e**) Normalized read count per gRNA of the selected hits in all samples for all libraries. (**f**) ICS analysis of eGFP-WT-RBM20 localization based on DRAQ5:RBM20 correlation upon indicated single gene knockouts. (**g**) Fluorescent microscopy analysis of eGFP-WT-RBM20 localization upon indicated single gene knockouts (n=3 technical replicates).

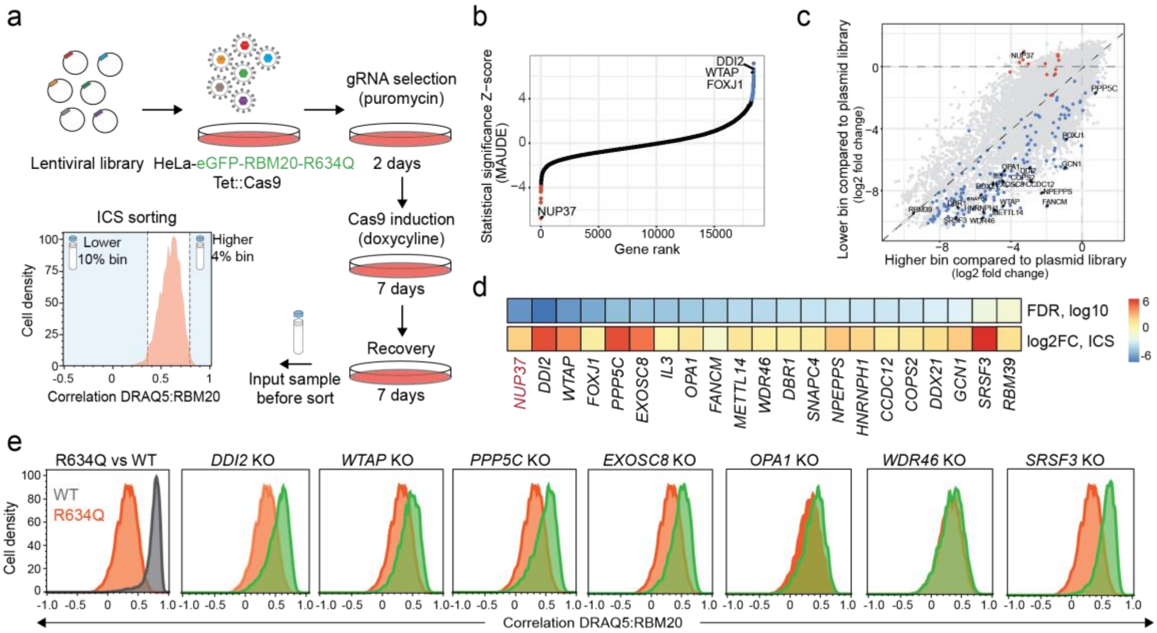


**Supplementary Figure 6. Genome-wide ICS screen with RBM20-R634Q to identify factors that retain RBM20-R634Q variant in the cytoplasm.** (**a**) Schematic outline of the ICS-screen. Five genome-wide lentiviral libraries[4] were applied to HeLa cells expressing eGFP-RBM20-R634Q and Tet::Cas9, with the coverage of 100 cells per gRNA. Cells were sorted based on the correlation between RBM20 and DRAQ5 into 4 % higher and 10 % lower fractions at final coverage of 500 cells per gRNA per sorted bin. Unsorted input samples were collected for sequencing as well. (**b**) Reads from collected samples were combined *in silico* to one dataset, with an average of 500 cells per gRNA, 5 gRNAs per gene. Hits were called using the software MAUDE[6]. Genes are ranked by their statistical significance. The horizontal dashed lines indicate an FDR of 1 %. Positive/negative regulators with FDR <1 % are marked in red and blue, respectively. (**c**) Scatter plot of fold changes visualizing gRNA abundance changes in higher (x axis) and lower (y axis) sorted bins compared with the plasmid library. Red and blue dots indicate statistically significant positive and negative regulators, respectively (FDR < 5 % according to MAUDE). Labelled are regulators selected for future analyses. (**d**) The impact of single knockouts of the selected hits from the genome-wide screen (one gRNA per gene; we picked the gRNA that showed the strongest Z-score in the pooled genetic screen) on RBM20 localization tested with ICS. The top row in the heatmap shows the log10 of FDR value for each candidate from the screen. The phenotype in the second row represents the standardized difference in RBM20 localization signal between the knockout (KO) and control (R634Q) cell populations (log2 of the ratio between cell fraction with R value for DRAQ5:RBM20 correlation > 0.7 in the knockout divided by cell fraction with R value for DRAQ5:RBM20 correlation > in the control). (**e**) ICS analysis of eGFP-RBM20-R634Q localization based on DRAQ5:RBM20 correlation upon indicated single gene knockouts.

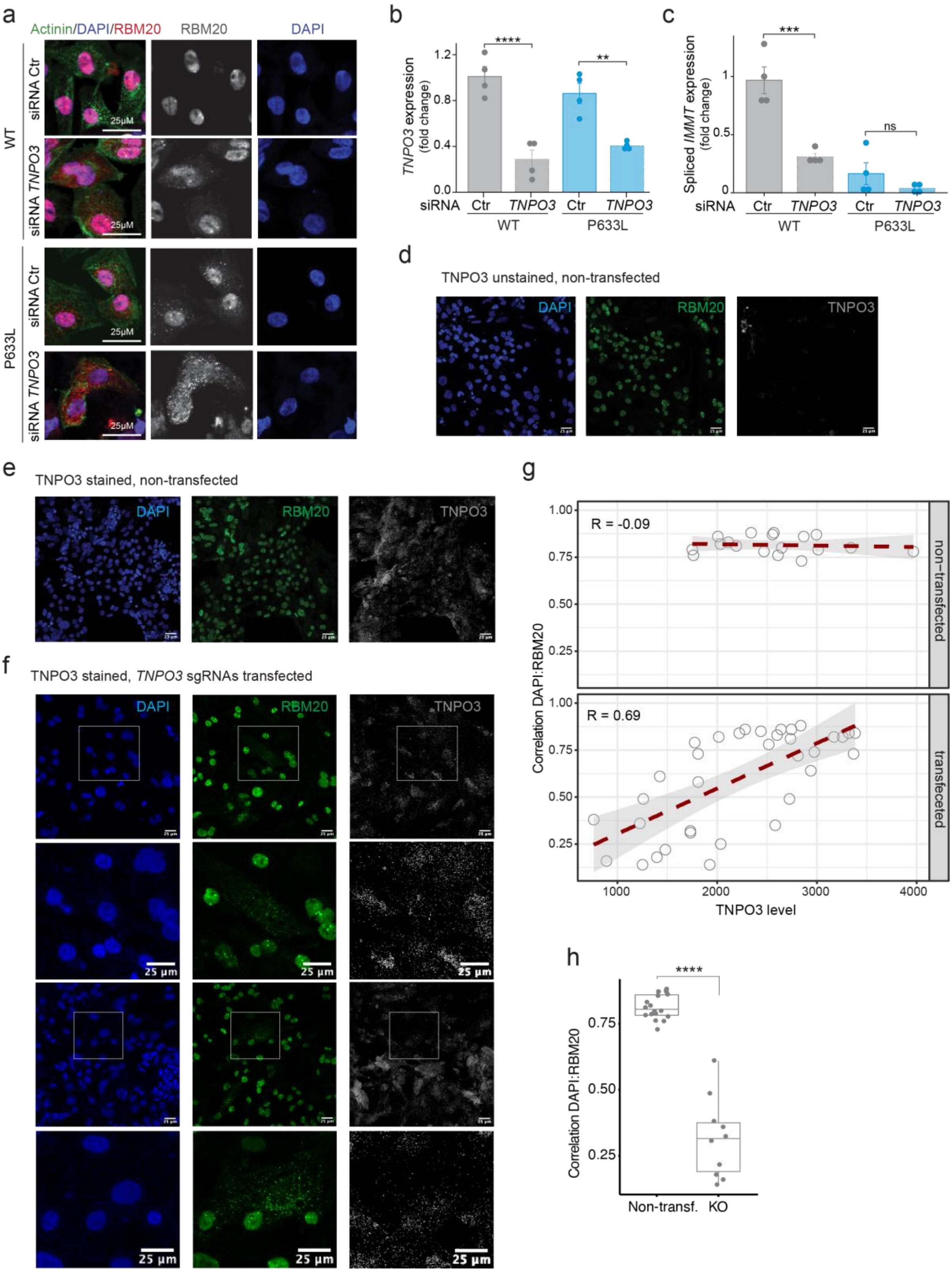


**Supplementary Figure 7. Validation of *TNPO3* perturbation effects on endogenous RBM20 mislocalization in iPSC-CMs.** (**a**) Representative confocal microscopy images of RBM20-WT or -P633L iPSC-CMs transfected with either control (Ctr) or targeting *TNPO3* siRNA (n=3). (**b**) qPCR analysis of *TNPO3* and (**c**) *IMMT* exon 6 spliced out isoform expression in RBM20-WT or -P633L iPSC-CMs transfected with either control or targeting *TNPO3* siRNA. Data are normalized to *GAPDH* expression and displayed as mean fold change versus one of the RBM20-WT transfected with control siRNA line, standard errors are indicated. Two biological replicates with

two technical replicates for each of them were analyzed. Ns = not significant, * - $p < 0.05$, ** - $p < 0.01$, *** - p < 0.001, **** - $p < 0.0001$ one-way ANOVA with Tukey's HSD post-test, two-tailored. (**d**) A representative image of iPSC-CMs stably expressing Cas9, not transfected with sgRNAs, and not stained with anti-TNPO3 antibody (n=3 technical replicates). (**e**) A representative image of iPSC-CMs stably expressing Cas9, not transfected with sgRNAs, stained with anti-TNPO3 antibody (n=3 technical replicates). (**f**) Representative images of iPSC-CMs stably expressing Cas9, transfected with a mix of three sgRNAs targeting *TNPO3*, stained with anti-TNPO3 antibody (n=3 technical replicates). (**g**) Correlation between DAPI:RBM20 co-localization and TNPO3 expression in non-transfected and transfected with sgRNAs targeting *TNPO3* iPSC-CMs. Linear regressions between the two are displayed as a dashed line, and regression standard errors are displayed in grey. Correlation coefficient = -0.09, p-value = 0.71 for non-transfected cells, and correlation coefficient = 0.69, p-value = 4.79e-06 for transfected cells, Pearson's product-moment correlation. Each dot represents a single cell. (**h**) DAPI:RBM20 co-localization in non-transfected cells (average pixel intensity of TNPO3 staining > 1750, n = 18) and in transfected cells with average pixel intensity of TNPO3 staining < 1750 (referred as "KO., n = 10). **** - $p < 0.0001$ two-tailored Student's T-test. All boxplots display quartiles Q1, Q2 (center), and Q3, with whiskers extending to the furthest data point that is within 1.5 times the IQR.

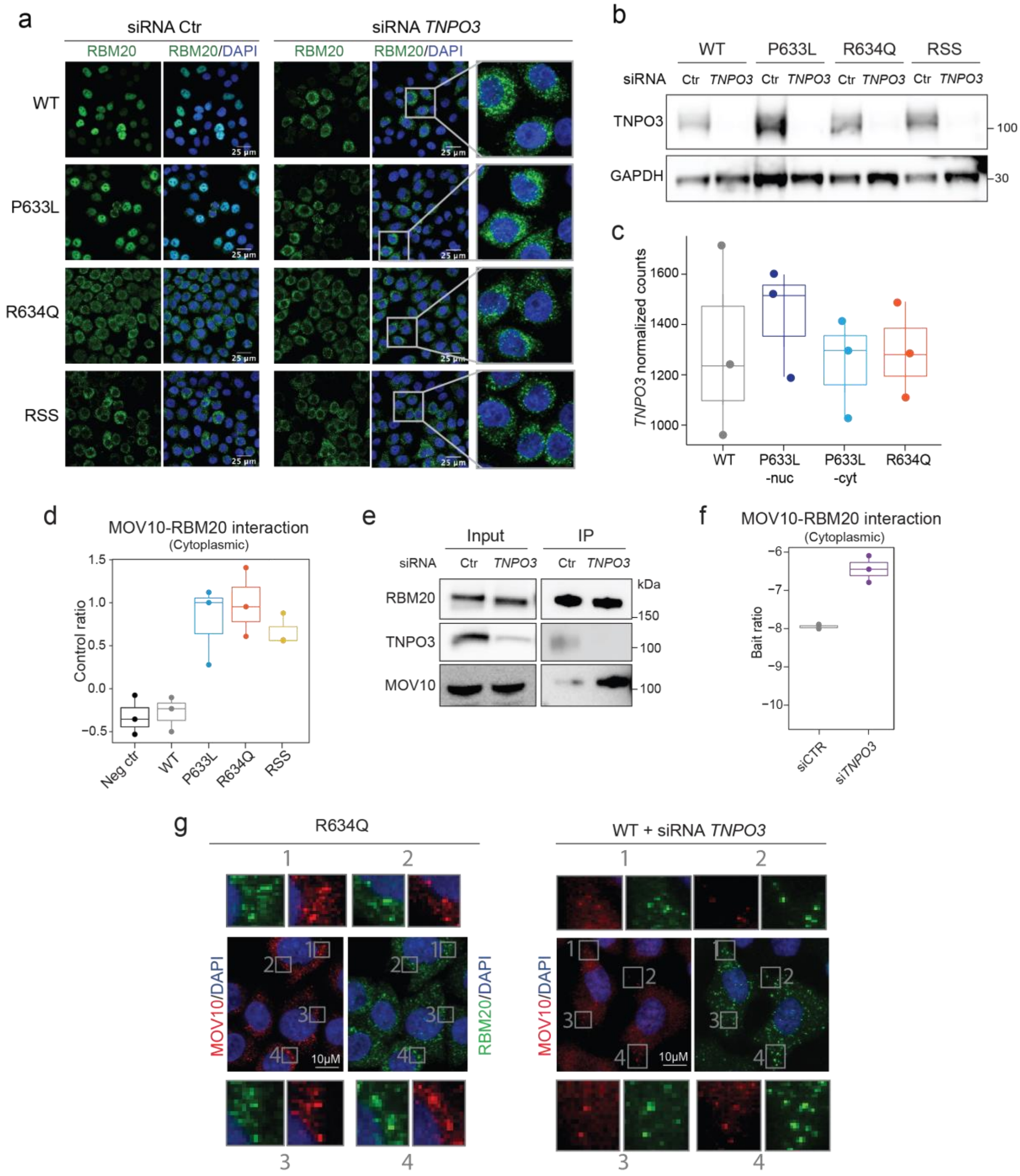


**Supplementary Figure 8. siRNA knockdown of *TNPO3* in HeLa-RBM20 reporter cells results in cytoplasmic granule formation of RBM20-WT.** (**a**) Representative confocal microscopy images of HeLa cell lines stably expressing the indicated eGFP-RBM20 variants transfected with either control (Ctr) or targeting *TNPO3* siRNA, n=3. (**b**) Representative Western blot analysis of TNPO3 and GAPDH levels in the HeLa reporter cell lines upon transfection with either control, or targeting *TNPO3* siRNA. (**c**) Normalized read counts mapping to *TNPO3* gene from RNA sequencing data of RBM20-WT, -P633L-nuc, -P633L-cyt, or -R634Q iPSC-CMs (n=3), sorting strategy described in the Fig. 1h. (**d**) Quantification of MOV10 peptides identified by mass-spectrometry in cytoplasmic fraction of co-immunoprecipitants with no-bait control, RBM20-WT, -P633L, -R634Q, or -RSS, normalized to the no-bait control (Neg ctrl). Three biological replicates. (**e**) Quantification of MOV10 peptides identified by mass-spectrometry in cytoplasmic fractions of co-immunoprecipitants with RBM20-WT in the presence or absence of TNPO3. Three biological replicates (n=3). (**f**) Representative Western blot of RBM20, TNPO3, and MOV10 in the cytoplasmic fraction of reporter HeLa cells expressing eGFP-

RBM20-WT in the presence or absence of TNPO3, as well as in the co-immunoprecipitant with RBM20 (from n=3 biological replicates). (**g**) Representative confocal microscopy images of HeLa cell lines stably expressing the indicated eGFP-RBM20 variants transfected with either control or targeting *TNPO3* siRNA, stained with antibodies against MOV10 (n=3 technical replicates). Each boxplot of this figure (panels c, d, f) displays quartiles Q1, Q2 (center), and Q3, with whiskers extending to the furthest data point that is within 1.5 times the IQR. * - $p < 0.05$, ** - $p < 0.01$, *** - $p < 0.001$, **** - $p < 0.0001$, one-way ANOVA with Tukey's HSD post-test.

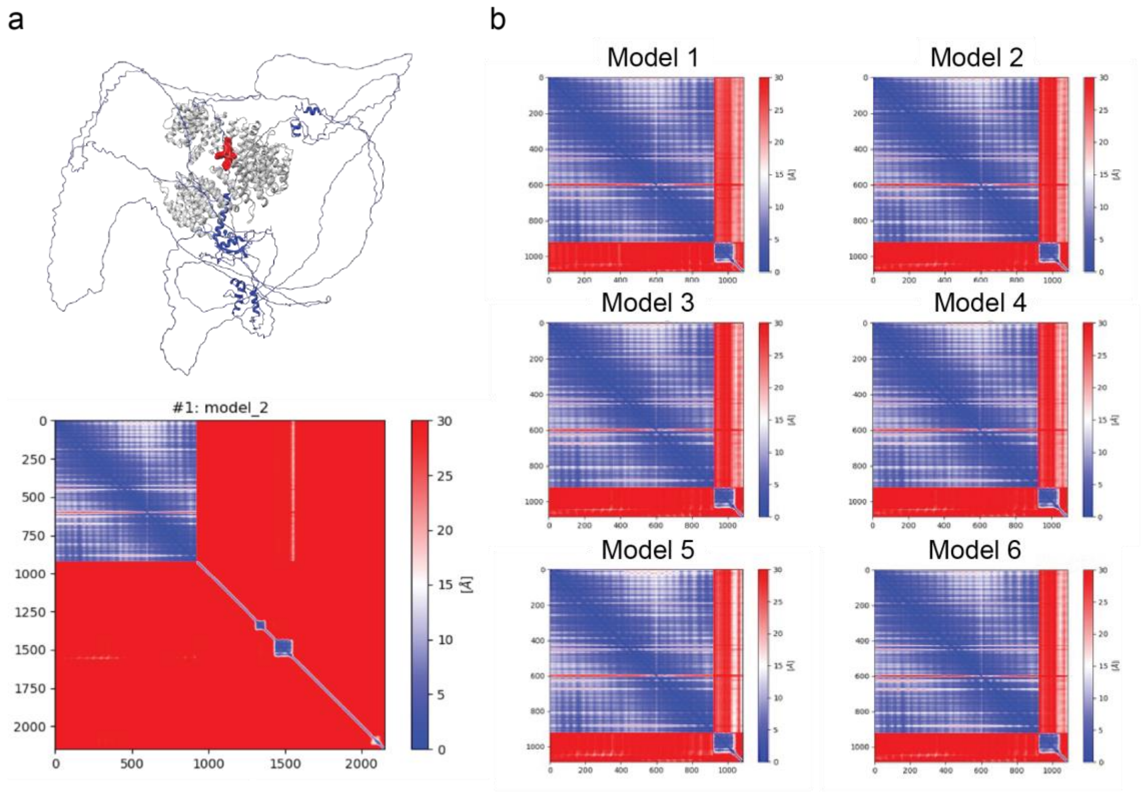


**Supplementary Figure 9. AlphaFold prediction of the RBM20-TNPO3 complex.** (**a**) Representative AlphaFold2[7] model of full-length RBM20 (blue, amino acid 1-1227) in complex with TNPO3 (grey, full length, amino acid 1-923). The known PRSRSP motif from amino acid 633 - 638 in RBM20 is highlighted as red spheres. Predicted Aligned Error (PAE) plot is shown below the model. (**b**) Predicted Aligned Error (PAE) plots for 6 representative AlphaFold models of the RBM20's RRM-RS domain in complex with TNPO3. Blue indicates a high confidence and red a low confidence in the relative position of pairs of residues.

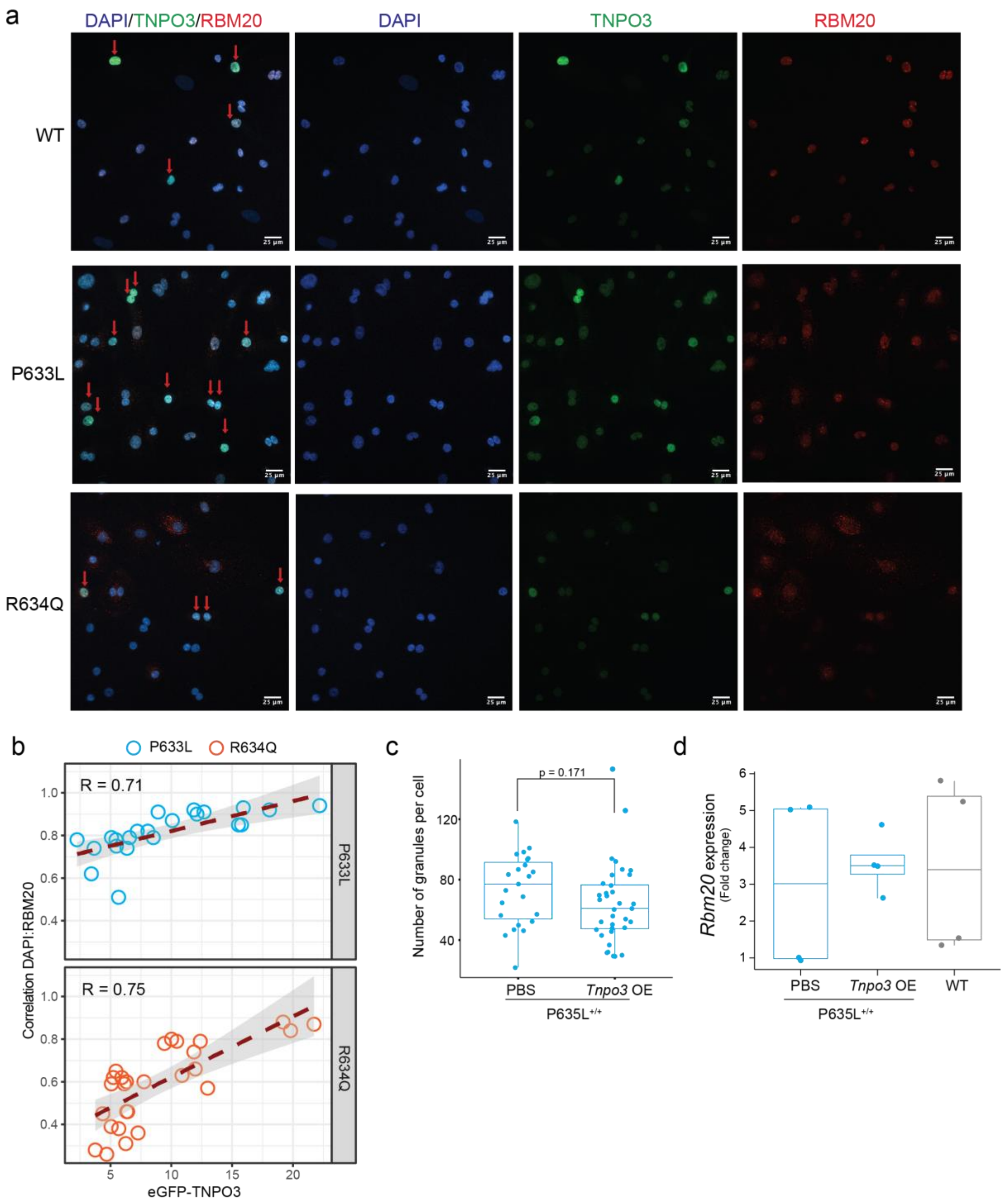


**Supplementary Figure 10. Overexpression of TNPO3 in iPSC-CMs and mice** (**a**) Representative confocal microscopy images of iPSC-CMs with RBM20-WT, -P633L, or -R634Q stained with an RBM20 antibody transduced with eGFP-tagged *TNPO3*. Red arrows point at cells transduced with eGFP-TNPO3, n=3. (**b**) Correlation between nuclear localization of RBM20 (DAPI:RBM20 correlation) and eGFP-TNPO3 fluorescence level (average pixel intensity), based on microscopy data from panel (a) for RBM20-P633L and -R634Q iPSC-CMs transduced with eGFP-TNPO3. Each dot represents a single cell. (**c**) Number of RBM20 granules per cell, primary cardiomyocytes isolated from n=4 mice treated with wither PBS or AAV9 delivering *Tnpo3* cDNA, four weeks post injection (p value = 0.171, two-tailored t-test). (**d**) qPCR analysis of *Rbm20* expression in n=4 WT mice, n=4 P635L$^{+/+}$ mice injected with PBS, or n=4 P635L$^{+/+}$ mice injected with *Tnpo3*. Data is normalized to *Gapdh* expression and displayed as fold change versus one of the WT mice with standard errors indicated. Each

boxplot of this figure (panels c, d) displays quartiles Q1, Q2 (center), and Q3, with whiskers extending to the furthest data point that is within 1.5 times the IQR.

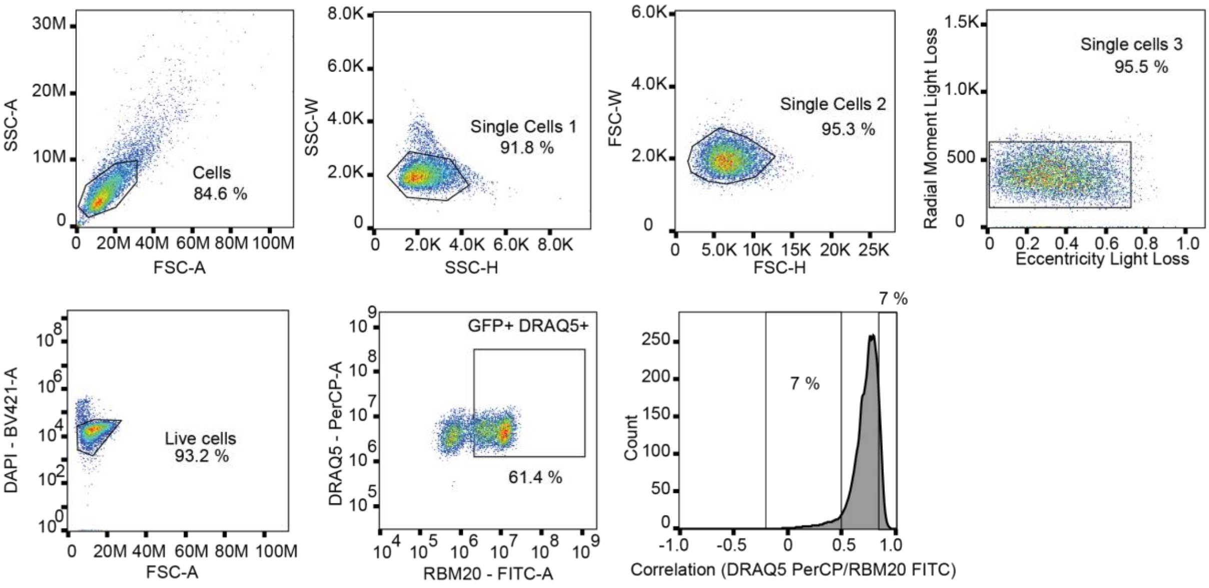


**Supplementary Figure 11. Gating scheme for HeLa Tet::Cas9 eGFP-RBM20-WT cells used for genome-wide ICS CRISPR screen.** Same strategy was used for all other ICS experiments, with exact gates for the last gate Correlation (DRAQ5:RBM20) specified within main text and/or methods. For FACS sorting, the same gating strategy was used, except for image-based gates (Eccentricity Light Loss vs Radial Moment Light Loss, and Correlation (DRAQ5:RBM20)).

**Supplementary Table 1.** Cloning oligonucleotides

| Name | Forward primer | Reverse primer |
|---|---|---|
| TetO-eGFP-GGSG-Flag-RBM20: eGFP cDNA | agcctccgcggccccgaattcgccaccatggtgagcaagg | ttgtagtcaccagaaccacccttgtacagctcgtccatgc |
| TetO-eGFP-GGSG-Flag-RBM20: Flag-RBM20 cDNA | gggtggttctggtgactacaaggacgacgatgacaaggtg | atagtcttctctagaggatcctcagagctttttcctttcg |
| TetO-eGFP-GGSG-Flag-RBM20: TetO-lenti backbone | gatcctctagagaagactat | aattcggggccgcggaggct |
| N-NLS-tag site directed mutagenesis | ccaaaaaagaagagaaaggtagactacaaggacgacgatga | tacctttctcttcttttttggaccagaacccacccttgtaca |
| P633L site directed mutagenesis | tatggcccagaaaggctgcggtctcgtagtccg | cggactacgagaccgcagcctttctgggccata |
| R634Q site directed mutagenesis | gcccagaaaggccgcagtctcgtagtccggt | accggactacgagactgcggcctttctgggc |
| R634Q-S635E site directed mutagenesis | ccagaaaggccgcaggaacgtagtccggtgagc | gctcaccggactacgttcctgcggcctttctgg |
| R634Q-S635E-S637E site directed mutagenesis | aggccgcaggaacgtgaaccggtgagccggtca | tgaccggctcaccggttcacgttcctgcggcct |
| pEFa-eGFP-RBM20: eGFP-GSSG-RBM20-cDNA | ggtgtcgtgacgcgggatccgccaccatggtgagcaaggg | atagtcttctctagaggatcctcagagctttttcctttcg |
| pEFa-eGFP-RBM20: backbone from the TetO-plasmids | gatcctctagagaagactat | cactcgagccggcgccaaagtggatctctgctgtccctgt |
| pEFa-eGFP-RBM20: promoter | acagggacagcagagatccactttggcgccggctcgagtg | cccttgctcaccatggtggcggatcccgcgtcacgacacc |
| AAV9-mTNPO3: TNPO3 cDNA | tcactttttttcaggttggaggtaccatggagggagccaa | cagaggttgattatcagatcggatccctatcgaaacaacc |

**Supplementary Table 2.** sgRNA cloning oligonucleotides

| Target gene | Forward | Reverse |
|---|---|---|
| TNPO3 | CACCGGGGATGTGTGCAAACACTGG | AAACCCAGTGTTTGCACACATCCCC |
| CLDN14 | CACCGCGATTGTCTTTGTAGGCAGC | AAACGCTGCCTACAAAGACAATCGC |
| ADAMTS16 | CACCGGCAGGCACCTGCGTGCGCCA | AAACTGGCGCACGCAGGTGCCTGCC |
| GALE | CACCGGAGAAGGTGCTGGTAACAGG | AAACCCTGTTACCAGCACCTTCTCC |
| SLC29A2 | CACCGGGGCGTGATAAAGTACCCCA | AAACTGGGGTACTTTATCACGCCCC |
| CEBPB | CACCGATGCTGGGTCCCAGGCCACC | AAACGGTGGCCTGGGACCCAGCATC |
| UBQLNL | CACCGGAGTGGCACAGATATGGCTC | AAACGAGCCATATCTGTGCCACTCC |
| TRIM33 | CACCGGACACACGGCGCAGGTGTCC | AAACGGACACCTGCGCCGTGTGTCC |
| PMM2 | CACCGATCGGACTTTGAGAAAGTGC | AAACGCACTTTCTCAAAGTCCGATC |
| TRIM24 | CACCGGAGGACAACGCAGAAGCCAA | AAACTTGGCTTCTGCGTTGTCCTCC |
| IPPK | CACCGCAAAGATGCCCGGAGCCCCG | AAACCGGGGCTCCGGGCATCTTTGC |

| | | |
|---|---|---|
| TTN | CACCGGTGCAGATCTCCTTTAGCGA | AAACTCGCTAAAGGAGATCTGCACC |
| XPO6 | CACCGCACCTTATCGACAAGTCGCA | AAACTGCGACTTGTCGATAAGGTGC |
| AKT2 | CACCGCATCGAGAGGACCTTCCACG | AAACCGTGGAAGGTCCTCTCGATGC |
| CLK1 | CACCGTACACTCAAGGATGTGAACC | AAACGGTTCACATCCTTGAGTGTAC |
| SPRK1 | CACCGATGGAGAAAGAGTCGGGCCC | AAACGGGCCCGACTCTTTCTCCATC |
| LMNA | CACCGCCATGGAGACCCCGTCCCAG | AAACCTGGGACGGGGTCTCCATGGC |
| CCDC12 | CACCGACTCACCACCTCCTCGATGA | AAACTCATCGAGGAGGTGGTGAGTC |
| CEBPB | CACCGATGCTGGGTCCCAGGCCACC | AAACGGTGGCCTGGGACCCAGCATC |
| COPS2 | CACCGGGCCAAGATGTCTGACATGG | AAACCCATGTCAGACATCTTGGCCC |
| DBR1 | CACCGAAGAGTTACCCTATGGTGGC | AAACGCCACCATAGGGTAACTCTTC |
| DDI2 | CACCGGTGTAACATAATGAGACTGG | AAACCCAGTCTCATTATGTTACACC |
| DDX21 | CACCGCCATCATGTTTACAGCGGGA | AAACTCCCGCTGTAAACATGATGGC |
| EXOSC8 | CACCGAATCTCCTGTAATACTCCAG | AAACCTGGAGTATTACAGGAGATTC |
| FANCM | CACCGGAAATTGTACATGACCACGG | AAACCCGTGGTCATGTACAATTTCC |
| FOXJ1 | CACCGGAGAAGGACGAACCAGGCAA | AAACTTGCCTGGTTCGTCCTTCTCC |
| GCN1 | CACCGGGTCCGGCGACAGGACGGAA | AAACTTCCGTCCTGTCGCCGGACC |
| GLDN14 | CACCGCGATTGTCTTTGTAGGCAGC | AAACGCTGCCTACAAAGACAATCGC |
| HNRNPH | CACCGGCTCGGCCGATGAAGTGCAG | AAACCTGCACTTCATCGGCCGAGCC |
| IL3 | CACCGGGCGGACCAGGAGTTGGAGC | AAACGCTCCAACTCCTGGTCCGCCC |
| METTL14 | CACCGGGGGTTGGACCTTGGAAGAG | AAACCTCTTCCAAGGTCCAACCCCC |
| NPEPPS | CACCGTTGTGATAGGGACCATCCAC | AAACGTGGATGGTCCCTATCACAAC |
| OPA1 | CACCGCAAGCTCGAATATTCCCAAG | AAACCTTGGGAATATTCGAGCTTGC |
| PPP5C | CACCGCATCAAGGGTTATTACCGCC | AAACGGCGGTAATAACCCTTGATGC |
| RBM39 | CACCGTGGCGGCAAGAATTCGACCA | AAACTGGTCGAATTCTTGCCGCCAC |
| SLSF3 | CACCGGCAACCCACACACTTCGGAG | AAACCTCCGAAGTGTGTGGGTTGCC |
| SNAPC4 | CACCGGACACTGGGAGCCAGACCGA | AAACTCGGTCTGGCTCCCAGTGTCC |
| WDR46 | CACCGTATGTGCGAGATCAACGTCA | AAACTGACGTTGATCTCGCACATAC |
| WTAP | CACCGACAGCAGGAGTCTGCACGCA | AAACTGCGTGCAGACTCCTGCTGTC |
| TNPO3-KO-1 | CACCGAGTCCGAAGCAGCTTCATGT | AAACACATGAAGCTGCTTCGGACTC |
| TNPO3-KO-2 | CACCGGAAAGAAGGCAACCCACCCT | AAACAGGGTGGGTTGCCTTCTTTCC |
| TNPO3-KO-3 | CACCGAAGGCGATCTAAGAACACTG | AAACCAGTGTTCTTAGATCGCCTTC |

**Supplementary Table 3.** Antibodies used. WB = Western Blot, IF = Immunofluorescence, ICS = image-enabled cell sorting.

| Antibody | Brand and reference | Dilution, Use |
|---|---|---|
| anti-TNPO3 | Invitrogen MA5-37991 | 1:1000, WB |
| anti-GAPDH | Abcam ab9485 | 1:1000, WB |
| anti-MOV10 | Sigma PLA0195 | 1:1000, WB;<br>1:250, IF |
| anti-RBM20 | Abcam ab233147 | 1:1000, WB;<br>1:250 IF;<br>1:100 ICS |
| anti-H3 | Abcam ab176842 | 1:1000, WB |
| anti-RBM20 | Invitrogen PA5-58068 | 1:250, IF |
| anti-alpha-Actinin | Abcam ab9465 | 1:250, IF |
| anti-TNPO3 | Abcam ab54353 | 1:250, IF |
| Goat-anti-rabbit-HRP | Abcam ab97051 | 1:10,000, WB |
| AlexaFluor488 goat-anti-rabbit | Invitrogen A32731 | 1:500, IF, ICS |
| AlexaFluor488 goat-anti-mouse | Invitrogen A11001 | 1:500, IF |
| AlexaFluor568 goat-anti-rabbit | Invitrogen A11011 | 1:500, IF |
| AlexaFluor568 goat-anti-mouse | Invitrogen A11004 | 1:500, IF |

**Supplementary Table 4.** qPCR oligonucleotides

| Name | Forward | Reverse |
|---|---|---|
| CMV promoter - AAV9 titration | gtaacgccaatagggactttcc | gggcgtacttggcatatgatac |
| hsGAPDH | tcgacagtcagccgcatctt | ctccgaccttcaccttcccc |
| hsTTN spliced-in | aattcaactgggggttctttcac | ctgagggatgtaaagttagaagatgc |
| hsTTN spliced-out | gaattccacatgaggagctttcac | ctgagggatgtaaagttagaagatgc |
| hsRBM20 | cagagggagagggacatgttcc | ggagggctgtgggaagagctgc |
| hsTNPO3 | agcaagtcactagtgctgagg | cagtgcaggagtgtgagcta |
| hsIMMT spliced-in | acctgcactttcagaagaagc | ttttcctgttgtgcaaggcg |
| hsIMMT spliced-out | tgcactttcagctctagcca | agaattgtccatggcggctt |
| mRbm20 | tccattcccagagggagaggga | gagaggggcctgggggac |
| mTnpo3 | gagagtgtcagacctggtgaag | agagaaccgcttctgtcacctc |
| mGapdh | aacagcaactcccactcttc | cctgttgctgtagccgtatt |
| mTtn unspliced | cagaaggaagagttcacacgc | aaccgggggttctttcac |
| mTtn spliced | cagaaggaagagttcacacgc | attccacatgaggagctttcac |
| mTtn RT-PCR | gtccacgaggaatgggagga | ttgtcacaggaacaggaatc |

**Supplementary Table 5.** CROPseq sequencing library oligonucleotides

| Name | Sequence |
|---|---|
| pU6 fwd | gggcctatttcccatgattccttc |
| pLTR-CROP-rev | cagatctggtctaaccagagagacccag |
| CROPseq_libQC_i5_s1 | aatgatacggcgaccaccgagatctacacactctttccctacacgacgctcttccgatcttcttgtggaaaggacgaaacaccg |
| CROPseq_libQC_i5_s2 | aatgatacggcgaccaccgagatctacacactctttccctacacgacgctcttccgatctatcttgtggaaaggacgaaacaccg |
| CROPseq_libQC_i5_s3 | aatgatacggcgaccaccgagatctacacactctttccctacacgacgctcttccgatctgatcttgtggaaaggacgaaacaccg |
| CROPseq_libQC_i5_s4 | aatgatacggcgaccaccgagatctacacactctttccctacacgacgctcttccgatctcgatcttgtggaaaggacgaaacaccg |
| CROPseq_libQC_i5_s5 | aatgatacggcgaccaccgagatctacacactctttccctacacgacgctcttccgatcttcgatcttgtggaaaggacgaaacaccg |
| CROPseq_libQC_i5_s6 | aatgatacggcgaccaccgagatctacacactctttccctacacgacgctcttccgatctatcgatcttgtggaaaggacgaaacaccg |
| CROPseq_i7:1 | caagcagaagacggcatacgagattcgccttagtgactggagttcagacgtgtgctcttccgatcgtgtctcaagatctagttacgccaagc |
| CROPseq_i7:2 | caagcagaagacggcatacgagatctagtacggtgactggagttcagacgtgtgctcttccgatcgtgtctcaagatctagttacgccaagc |
| CROPseq_i7:3 | caagcagaagacggcatacgagatttctgcctgtgactggagttcagacgtgtgctcttccgatcgtgtctcaagatctagttacgccaagc |
| CROPseq_i7:4 | caagcagaagacggcatacgagatgctcaggagtgactggagttcagacgtgtgctcttccgatcgtgtctcaagatctagttacgccaagc |
| CROPseq_i7:5 | caagcagaagacggcatacgagataggagtccgtgactggagttcagacgtgtgctcttccgatcgtgtctcaagatctagttacgccaagc |
| CROPseq_i7:6 | caagcagaagacggcatacgagatcatgcctagtgactggagttcagacgtgtgctcttccgatcgtgtctcaagatctagttacgccaagc |
| CROPseq_i7:7 | caagcagaagacggcatacgagatgtagagaggtgactggagttcagacgtgtgctcttccgatcgtgtctcaagatctagttacgccaagc |
| CROPseq_i7:8 | caagcagaagacggcatacgagatcctctctggtgactggagttcagacgtgtgctcttccgatcgtgtctcaagatctagttacgccaagc |
| CROPseq_i7:9 | caagcagaagacggcatacgagatagcgtagcgtgactggagttcagacgtgtgctcttccgatcgtgtctcaagatctagttacgccaagc |
| CROPseq_i7:10 | caagcagaagacggcatacgagatcagcctcggtgactggagttcagacgtgtgctcttccgatcgtgtctcaagatctagttacgccaagc |
| CROPseq_i7:11 | caagcagaagacggcatacgagattgcctcttgtgactggagttcagacgtgtgctcttccgatcgtgtctcaagatctagttacgccaagc |
| CROPseq_i7:12 | caagcagaagacggcatacgagattcctctacgtgactggagttcagacgtgtgctcttccgatcgtgtctcaagatctagttacgccaagc |
| CROPseq_i7:13 | caagcagaagacggcatacgagattcatgagcgtgactggagttcagacgtgtgctcttccgatcgtgtctcaagatctagttacgccaagc |
| CROPseq_i7:14 | caagcagaagacggcatacgagatcctgagatgtgactggagttcagacgtgtgctcttccgatcgtgtctcaagatctagttacgccaagc |
| CROPseq_i7:15 | caagcagaagacggcatacgagattagcgagtgtgactggagttcagacgtgtgctcttccgatcgtgtctcaagatctagttacgccaagc |
| CROPseq_i7:16 | caagcagaagacggcatacgagatgtagctccgtgactggagttcagacgtgtgctcttccgatcgtgtctcaagatctagttacgccaagc |
| CROPseq_i7:17 | caagcagaagacggcatacgagattactacgcgtgactggagttcagacgtgtgctcttccgatcgtgtctcaagatctagttacgccaagc |
| CROPseq_i7:18 | caagcagaagacggcatacgagataggctccggtgactggagttcagacgtgtgctcttccgatcgtgtctcaagatctagttacgccaagc |

## References


1. Zhou, Y. *et al.* Metascape provides a biologist-oriented resource for the analysis of systems-level datasets. *Nat. Commun.* **10**, (2019).
2. Love, M. I., Huber, W. & Anders, S. Moderated estimation of fold change and dispersion for RNA-seq data with DESeq2. *Genome Biol.* **15**, 1–21 (2014).
3. Shen, S. *et al.* MATS: A Bayesian framework for flexible detection of differential alternative splicing from RNA-Seq data. *Nucleic Acids Res.* **40**, 1–13 (2012).
4. Schraivogel, D. *et al.* High-speed fluorescence image – enabled cell sorting. *Science (80-. ).* **320**, 315–320 (2022).
5. Hart, T. *et al.* Evaluation and design of genome-wide CRISPR/SpCas9 knockout screens. *G3 Genes, Genomes, Genet.* **7**, 2719–2727 (2017).
6. De Boer, C. G., Ray, J. P., Hacohen, N. & Regev, A. MAUDE: Inferring expression changes in sorting-based CRISPR screens. *Genome Biol.* **21**, 1–16 (2020).
7. Jumper, J. *et al.* Highly accurate protein structure prediction with AlphaFold. *Nature* **596**, 583–589 (2021).
