# Supplementary material for: Mislocalization of pathogenic RBM20 variants in dilated cardiomyopathy is caused by loss-of-interaction with Transportin-3
Source: Nat Commun. 2023 Jul 18;14:4312. doi: 10.1038/s41467-023-39965-6 (PMC10353998; doi:10.1038/s41467-023-39965-6)
Supplement: Supplementary file 3 — Description of Additional Supplementary Files [file 41467_2023_39965_MOESM3_ESM.pdf]

Description of Additional Supplementary Files

File Name: Supplementary Data 1
Description: Differentially expressed genes (DeSeq2), pairwise comparisons to RBM20-WT iPSC-CMs, Fig. 1i.

File Name: Supplementary Data 2
Description: All enrichment analyses done with Metascape, excel sheets labelled with respective figure panels.

File Name: Supplementary Data 3
Description: PSI values calculated with the pipeline described in Schafer et al., 2015. All exonic parts are annotated to exons with genomic coordinates indicated.

File Name: Supplementary Data 4
Description: calculated with rMATS statistics for each exon genome-wide, pairwise comparisons to WT. Each sheet has an rMATS output for an indicated pairwise comparison for an indicated event type, used for Supplementary Figure 2 g-h.

File Name: Supplementary Data 5
Description: calculated with rMATS overlaps with Briganti et al., 2020.

File Name: Supplementary Data 6
Description: Mass spectrometry hits (Fig. 1l, Supplementary Fig. 3)

File Name: Supplementary Data 7
Description: Differentially expressed genes (DeSeq2), pairwise comparisons to RBM20-WT expressing iPSC-CMs, Fig. 2d.

File Name: Supplementary Data 8
Description: PSI values calculated with the pipeline described in Schafer et al., 2015. All exonic parts are annotated to exons with genomic coordinates indicated.

File Name: Supplementary Data 9
Description: calculated with rMATS statistics for each exon genome-wide, pairwise comparisons to WT. Each sheet has an rMATS output for an indicated pairwise comparison for indicated event type, used for Supplementary Figure 4 g-h.

File Name: Supplementary Data 10
Description: gRNA and gene statistics from RBM20-WT ICS screen, Fig. 3, Supplementary Fig. 5.

File Name: Supplementary Data 11
Description: gRNA and gene statistics from RBM20-R634Q ICS screen, Supplementary Fig. 6.

File Name: Supplementary Data 12

Description: Normalized (DeSeq2) read counts for hits in HeLa and iPSC-CMs

File Name: Supplementary Data 13
Description: Raw read counts for HeLa and iPSC-CMs

File Name: Supplementary Data 14
Description: Plasmid sequences
