# Supplementary material for: Mislocalization of pathogenic RBM20 variants in dilated cardiomyopathy is caused by loss-of-interaction with Transportin-3
Source: Nat Commun. 2023 Jul 18;14:4312. doi: 10.1038/s41467-023-39965-6 (PMC10353998; doi:10.1038/s41467-023-39965-6)
Supplement: Supplementary file 17 — Supplementary Data 14 [file 41467_2023_39965_MOESM17_ESM.docx]

Sequences

>sp|P2147_GFPflagRBM20WT

mvskgeelftgvvpilveldgdvnghkfsvsgegegdatygkltlkficttgklpvpwptlvttltygvqcfsrypdhmkqhdffksampegyvqertiffkddgnyktraevkfegdtlvnrielkgidfkedgnilghkleynynshnvyimadkqkngikvnfkirhniedgsvqladhyqqntpigdgpvllpdnhylstqsalskdpnekrdhmvllefvtaagitlgmdelykggsgdykddddkvlaaamsqdadpsgpeqpdrvacsvpgaraspapsgprgmqqpppppqpppppqaglpqiiqnaaklldknpfsvsnpnpllpspaslqlaqlqaqltlhrlklaqtavtnntaaatvlnqvlskvamsqplfnqlrhpsvitgphghagvpqhaaaipstrfpsnaiafsppsqtrgpgpsmnlpnqppsamvmhpftgvmpqtpgqpavilgigktgpapatagfyeygkassgqtygpetdgqpgflpssastsgsvtyeghyshtgqdgqaafskdfygpnsqgshvasgfpaeqagglksevgpllqgtnsqwesphgfsgqskpdltagpmwppphnqpyelydpeeptsdrtppsfggrlnnskqgfigagrrakedqallsvrplqahelndfhgvaplhlphicsicdkkvfdlkdwelhvkgklhaqkclvfsenagircilgsaegtlcaspnstavynpagnedyasnlgtsyvpiparsftqssptfplasvgttfaqrkgagrvvhicnlpegsctendvinlglpfgkvtnyilmkstnqaflemayteaaqamvqyyqeksavingekllirmskrykelqlkkpgkavaaiiqdihsqrerdmfreadrygperprsrspvsrslsprshtpsftscssshsppgpsradwgngrdswehspyarreeerdpapwrdngddkrdrmdpwahdrkhhprqldkaelderpeggrphrekyprsgspnlphsvssyksredgyyrkepkaksdkylkqqqdapgrsrrkdearlresrhphpddsgkedglgpkvtrapegakakqneknktkrtdrdqegaddrkentmaeneagkeeqegmeespqsvgrqekeaefsdpentrtkkeqdweseseaegeswyptnmeelvtvdevgeeedfivepdipeleeivpidqkdkicpetclcvtttldldlaqdfpkegvkavgngaaeislksprelpsastscpsdmdvempglnldaerkpaesetglsledsdcyekeakgvessdvhpaptvqqmsspkpaeerarqpspfvddcktrgtpedgacegspleekasppietdlqnqacqevltpensryvemkslevrspeytevelkqplslpswepedvfselsiplgvefvvprtgfycklcglfytseetakmshcrsavhyrnlqkylsqlaeeglketegadsprpedsgivprferkkl

>TetO-FLAG-RBM20

cgcgttgacattgattattgactagttattaatagtaatcaattacggggtcattagttcatagcccatatatggagttccgcgttacataacttacggtaaatggcccgcctggctgaccgcccaacgacccccgcccattgacgtcaataatgacgtatgttcccatagtaacgccaatagggactttccattgacgtcaatgggtggagtatttacggtaaactgcccacttggcagtacatcaagtgtatcatatgccaagtacgccccctattgacgtcaatgacggtaaatggcccgcctggcattatgcccagtacatgaccttatgggactttcctacttggcagtacatctacgtattagtcatcgctattaccatggtgatgcggttttggcagtacatcaatgggcgtggatagcggtttgactcacggggatttccaagtctccaccccattgacgtcaatgggagtttgttttggcaccaaaatcaacgggactttccaaaatgtcgtaacaactccgccccattgacgcaaatgggcggtaggcgtgtacggtgggaggtctatataagcagcgcgttttgcctgtactgggtctctctggttagaccagatctgagcctgggagctctctggctaactagggaacccactgcttaagcctcaataaagcttgccttgagtgcttcaagtagtgtgtgcccgtctgttgtgtgactctggtaactagagatccctcagacccttttagtcagtgtggaaaatctctagcagtggcgcccgaacagggacttgaaagcgaaagggaaaccagaggagctctctcgacgcaggactcggcttgctgaagcgcgcacggcaagaggcgaggggcggcgactggtgagtacgccaaaaattttgactagcggaggctagaaggagagagatgggtgcgagagcgtcagtattaagcgggggagaattagatcgcgatgggaaaaaattcggttaaggccagggggaaagaaaaaatataaattaaaacatatagtatgggcaagcagggagctagaacgattcgcagttaatcctggcctgttagaaacatcagaaggctgtagacaaatactgggacagctacaaccatcccttcagacaggatcagaagaacttagatcattatataatacagtagcaaccctctattgtgtgcatcaaaggatagagataaaagacaccaaggaagctttagacaagatagaggaagagcaaaacaaaagtaagaccaccgcacagcaagcggccgctgatcttcagacctggaggaggagatatgagggacaattggagaagtgaattatataaatataaagtagtaaaaattgaaccattaggagtagcacccaccaaggcaaagagaagagtggtgcagagagaaaaaagagcagtgggaataggagctttgttccttgggttcttgggagcagcaggaagcactatgggcgcagcgtcaatgacgctgacggtacaggccagacaattattgtctggtatagtgcagcagcagaacaatttgctgagggctattgaggcgcaacagcatctgttgcaactcacagtctggggcatcaagcagctccaggcaagaatcctggctgtggaaagatacctaaaggatcaacagctcctggggatttggggttgctctggaaaactcatttgcaccactgctgtgccttggaatgctagttggagtaataaatctctggaacagatttggaatcacacgacctggatggagtgggacagagaaattaacaattacacaagcttaatacactccttaattgaagaatcgcaaaaccagcaagaaaagaatgaacaagaattattggaattagataaatgggcaagtttgtggaattggtttaacataacaaattggctgtggtatataaaattattcataatgatagtaggaggcttggtaggtttaagaatagtttttgctgtactttctatagtgaatagagttaggcagggatattcaccattatcgtttcagacccacctcccaaccccgaggggacccgacaggcccgaaggaatagaagaagaaggtggagagagagacagagacagatccattcgattagtgaacggatcggcactgcgtgcgccaattctgcagacaaatggcagtattcatccacaattttaaaagaaaaggggggattggggggtacagtgcaggggaaagaatagtagacataatagcaacagacatacaaactaaagaattacaaaaacaaattacaaaaattcaaaattttcgggtttattacagggacagcagagatccagtttggttagatctcgagtttaccactccctatcagtgatagagaaaagtgaaagtcgagtttaccactccctatcagtgatagagaaaagtgaaagtcgagtttaccactccctatcagtgatagagaaaagtgaaagtcgagtttaccactccctatcagtgatagagaaaagtgaaagtcgagtttaccactccctatcagtgatagagaaaagtgaaagtcgagtttaccactccctatcagtgatagagaaaagtgaaagtcgagtttaccactccctatcagtgatagagaaaagtgaaagtcgagctcggtacccgggtcgagtaggcgtgtacggtgggaggcctatataagcagagctcgtttagtgaaccgtcagatcgcctggagacgccatccacgctgttttgacctccatagaagacaccgggaccgatccagcctccgcggccccgaattcgccaccatggactacaaggacgacgatgacaaggtgctggcagcagccatgagccaggacgcggaccccagcggtccggagcagccggacagagttgcctgcagtgtgcctggtgcccgggcgtccccggcaccctccggcccgcgagggatgcagcagccgccgccgccgccccagccaccgcccccgccccaagccggcctaccccagatcatccaaaatgccgccaagctcctggacaagaacccattctcggtcagtaacccgaaccctctgcttccttcacctgccagtctccagctggctcaactgcaggcccagctcaccctccaccggctgaagctggcacagacagctgtcaccaacaacactgcagccgccacagtcctgaaccaagtcctctccaaagtggccatgtcccagcctctcttcaatcaactgaggcatccgtctgtgatcactggcccccacggccatgctggggttccccaacatgctgcagccatacccagcacgcgcttcccatcaaatgcaattgccttttcaccccccagccagacacgaggccccggaccctccatgaaccttcccaaccagccacccagtgccatggtgatgcatcctttcactggggtaatgcctcagacccctggccagccagcagtcatcttgggcattggcaagactgggcctgctccagctacagcaggattctatgagtatggcaaagccagctctggccagacatatggccctgaaacagatggtcagcctggcttcctgccatcctcggcctcaacctcgggcagtgtgacctatgaagggcactacagccacacagggcaggatggtcaagctgccttttccaaagatttttacggacccaactcccaaggttcacatgtggccagcggatttccagctgagcaggctgggggcctgaaaagtgaggtcgggccactgctgcagggcacaaacagccaatgggagagcccccatggattctcgggccaaagcaagcctgatctcacagcaggtcccatgtggcctccaccccacaaccagccctatgagctgtacgaccccgaggaaccaacctcagacaggacacctccttccttcgggggtcggcttaacaacagcaaacagggttttatcggtgctgggcggagggccaaggaggaccaggcgttgctatctgtgcggcccctgcaggctcatgagctgaacgactttcacggtgtggcccccctccacttgccgcatatctgtagcatctgtgacaagaaggtgtttgatttgaaggactgggagctgcatgtgaaaggcaaactccatgcacaaaagtgcctggtcttctctgaaaatgctggcatccggtgtatacttggttcggcagagggaacattgtgtgcttctcccaacagcacagctgtttataaccctgctgggaatgaagattatgcctcaaatcttggaacatcatacgtgcccattccagcaaggtcattcactcagtcaagccccacatttcctttggcttctgtggggacaacttttgcacagcggaaaggggctggccgtgtggtgcacatctgcaatctccctgaaggaagctgcactgagaatgacgtcattaacctggggctgccctttggaaaggtcactaattacatcctcatgaagtcgactaatcaggcctttttagagatggcttacacagaagctgcacaggccatggtccagtattatcaagaaaaatctgctgtgatcaatggtgagaagttgctcattcggatgtccaagagatacaaggaattgcagctcaagaaacccgggaaggccgtggctgccatcatccaggacatccattcccagagggagagggacatgttccgggaagcagacagatatggcccagaaaggccgcggtctcgtagtccggtgagccggtcactctccccgaggtcccacactcccagcttcacctcctgcagctcttcccacagccctccgggcccctcccgggctgactggggcaatggccgggactcctgggagcactctccctatgccaggagggaggaagagcgagacccggctccctggagggacaacggagatgacaagagggacaggatggacccctgggcacatgatcgcaaacaccacccccggcaactggacaaggctgagttggacgagcgaccagaaggagggaggccccaccgggagaagtacccgagatctgggtctcccaacctgccccactctgtgtccagctacaaaagccgtgaagacggctactaccggaaagagcccaaagccaagtcggacaagtatctgaagcagcagcaggatgcccccgggaggtccaggaggaaagacgaggccaggctgcgggaaagcagacacccccatccggatgactcaggcaaggaagatgggctggggccaaaggtcactagggcccctgagggcgccaaggccaagcagaatgagaaaaataaaaccaagagaactgatagagaccaagaaggagctgatgatagaaaagaaaacacaatggcagagaatgaggctggaaaagaggaacaggagggcatggaggagagtccacagtctgtgggcagacaggagaaagaagcagagttctctgatccggaaaacacaaggacaaagaaggaacaagattgggagagtgaaagtgaggcagagggggagagctggtatcccactaacatggaggagctggtgacagtggacgaggttggggaagaagaagattttatcgtggaaccagacatcccagagctggaagaaattgtgcccattgaccagaaagacaaaatttgcccagaaacatgtctgtgtgtgacaaccaccttagacttagacctggcccaggatttccccaaggaaggagtcaaggccgtagggaatggggctgcagaaatcagcctcaagtcacccagagaactgccctctgcttccacaagctgtcccagtgacatggacgtcgagatgccggggcttaacctggatgctgagcggaagccagctgaaagtgagacaggcctctccctggaggattcagattgctacgagaaggaggcaaagggagtggagagctcagatgttcatccagcccctacagtccagcaaatgtcttcccctaagccagcagaggagagggcccggcagccaagcccatttgtggatgattgcaagaccagggggacccccgaagatggggcttgtgaaggcagccccctggaggagaaagccagcccccccatcgaaactgacctccaaaaccaagcttgccaagaagtgttgaccccggaaaactccaggtacgtggaaatgaaatctctggaggtgaggtcaccagagtacactgaagtggaactgaaacagcccctttctttgccctcttgggaaccagaggatgtgttcagtgaacttagcattcctctaggggtggagttcgtggttcccaggactggcttttattgcaagctgtgtgggctgttctacacgagcgaggagacagcaaagatgagccactgccgcagcgctgtccactacaggaacttacagaaatatttgtcccagctggccgaggagggcctcaaggagaccgagggggcagatagcccgaggccagaggacagcggaatcgtgccacgcttcgaaaggaaaaagctctgaggatcctctagagaagactattaattaagctagctaattcgatatcaagcttatcgataatcaacctctggattacaaaatttgtgaaagattgactggtattcttaactatgttgctccttttacgctatgtggatacgctgctttaatgcctttgtatcatgctattgcttcccgtatggctttcattttctcctccttgtataaatcctggttgctgtctctttatgaggagttgtggcccgttgtcaggcaacgtggcgtggtgtgcactgtgtttgctgacgcaacccccactggttggggcattgccaccacctgtcagctcctttccgggactttcgctttccccctccctattgccacggcggaactcatcgccgcctgccttgcccgctgctggacaggggctcggctgttgggcactgacaattccgtggtgttgtcggggaaatcatcgtcctttccttggctgctcgcctgtgttgccacctggattctgcgcgggacgtccttctgctacgtcccttcggccctcaatccagcggaccttccttcccgcggcctgctgccggctctgcggcctcttccgcgtcttcgccttcgccctcagacgagtcggatctccctttgggccgcctccccgcatcgataccgtcgacctcgagacctagaaaaacatggagcaatcacaagtagcaatacagcagctaccaatgctgattgtgcctggctagaagcacaagaggaggaggaggtgggttttccagtcacacctcaggtacctttaagaccaatgacttacaaggcagctgtagatcttagccactttttaaaagaaaaggggggactggaagggctaattcactcccaacgaagacaagatatccttgatctgtggatctaccacacacaaggctacttccctgattggcagaactacacaccagggccagggatcagatatccactgacctttggatggtgctacaagctagtaccagttgagcaagagaaggtagaagaagccaatgaaggagagaacacccgcttgttacaccctgtgagcctgcatgggatggatgacccggagagagaagtattagagtggaggtttgacagccgcctagcatttcatcacatggcccgagagctgcatccggactgtactgggtctctctggttagaccagatctgagcctgggagctctctggctaactagggaacccactgcttaagcctcaataaagcttgccttgagtgcttcaagtagtgtgtgcccgtctgttgtgtgactctggtaactagagatccctcagacccttttagtcagtgtggaaaatctctagcagggcccgtttaaacccgctgatcagcctcgactgtgccttctagttgccagccatctgttgtttgcccctcccccgtgccttccttgaccctggaaggtgccactcccactgtcctttcctaataaaatgaggaaattgcatcgcattgtctgagtaggtgtcattctattctggggggtggggtggggcaggacagcaagggggaggattgggaagacaatagcaggcatgctggggatgcggtgggctctatggcttctgaggcggaaagaaccagctggggctctagggggtatccccacgcgccctgtagcggcgcattaagcgcggcgggtgtggtggttacgcgcagcgtgaccgctacacttgccagcgccctagcgcccgctcctttcgctttcttcccttcctttctcgccacgttcgccggctttccccgtcaagctctaaatcgggggctccctttagggttccgatttagtgctttacggcacctcgaccccaaaaaacttgattagggtgatggttcacgtagtgggccatcgccctgatagacggtttttcgccctttgacgttggagtccacgttctttaatagtggactcttgttccaaactggaacaacactcaaccctatctcggtctattcttttgatttataagggattttgccgatttcggcctattggttaaaaaatgagctgatttaacaaaaatttaacgcgaattaattctgtggaatgtgtgtcagttagggtgtggaaagtccccaggctccccagcaggcagaagtatgcaaagcatgcatctcaattagtcagcaaccaggtgtggaaagtccccaggctccccagcaggcagaagtatgcaaagcatgcatctcaattagtcagcaaccatagtcccgcccctaactccgcccatcccgcccctaactccgcccagttccgcccattctccgccccatggctgactaattttttttatttatgcagaggccgaggccgcctctgcctctgagctattccagaagtagtgaggaggcttttttggaggcctaggcttttgcaaaaagctcccgggagcttgtatatccattttcggatctgatcagcacgtgttgacaattaatcatcggcatagtatatcggcatagtataatacgacaaggtgaggaactaaaccatggccaagttgaccagtgccgttccggtgctcaccgcgcgcgacgtcgccggagcggtcgagttctggaccgaccggctcgggttctcccgggacttcgtggaggacgacttcgccggtgtggtccgggacgacgtgaccctgttcatcagcgcggtccaggaccaggtggtgccggacaacaccctggcctgggtgtgggtgcgcggcctggacgagctgtacgccgagtggtcggaggtcgtgtccacgaacttccgggacgcctccgggccggccatgaccgagatcggcgagcagccgtgggggcgggagttcgccctgcgcgacccggccggcaactgcgtgcacttcgtggccgaggagcaggactgacacgtgctacgagatttcgattccaccgccgccttctatgaaaggttgggcttcggaatcgttttccgggacgccggctggatgatcctccagcgcggggatctcatgctggagttcttcgcccaccccaacttgtttattgcagcttataatggttacaaataaagcaatagcatcacaaatttcacaaataaagcatttttttcactgcattctagttgtggtttgtccaaactcatcaatgtatcttatcatgtctgtataccgtcgacctctagctagagcttggcgtaatcatggtcatagctgtttcctgtgtgaaattgttatccgctcacaattccacacaacatacgagccggaagcataaagtgtaaagcctggggtgcctaatgagtgagctaactcacattaattgcgttgcgctcactgcccgctttccagtcgggaaacctgtcgtgccagctgcattaatgaatcggccaacgcgcggggagaggcggtttgcgtattgggcgctcttccgcttcctcgctcactgactcgctgcgctcggtcgttcggctgcggcgagcggtatcagctcactcaaaggcggtaatacggttatccacagaatcaggggataacgcaggaaagaacatgtgagcaaaaggccagcaaaaggccaggaaccgtaaaaaggccgcgttgctggcgtttttccataggctccgcccccctgacgagcatcacaaaaatcgacgctcaagtcagaggtggcgaaacccgacaggactataaagataccaggcgtttccccctggaagctccctcgtgcgctctcctgttccgaccctgccgcttaccggatacctgtccgcctttctcccttcgggaagcgtggcgctttctcatagctcacgctgtaggtatctcagttcggtgtaggtcgttcgctccaagctgggctgtgtgcacgaaccccccgttcagcccgaccgctgcgccttatccggtaactatcgtcttgagtccaacccggtaagacacgacttatcgccactggcagcagccactggtaacaggattagcagagcgaggtatgtaggcggtgctacagagttcttgaagtggtggcctaactacggctacactagaagaacagtatttggtatctgcgctctgctgaagccagttaccttcggaaaaagagttggtagctcttgatccggcaaacaaaccaccgctggtagcggtggtttttttgtttgcaagcagcagattacgcgcagaaaaaaaggatctcaagaagatcctttgatcttttctacggggtctgacgctcagtggaacgaaaactcacgttaagggattttggtcatgagattatcaaaaaggatcttcacctagatccttttaaattaaaaatgaagttttaaatcaatctaaagtatatatgagtaaacttggtctgacagttaccaatgcttaatcagtgaggcacctatctcagcgatctgtctatttcgttcatccatagttgcctgactccccgtcgtgtagataactacgatacgggagggcttaccatctggccccagtgctgcaatgataccgcgagacccacgctcaccggctccagatttatcagcaataaaccagccagccggaagggccgagcgcagaagtggtcctgcaactttatccgcctccatccagtctattaattgttgccgggaagctagagtaagtagttcgccagttaatagtttgcgcaacgttgttgccattgctacaggcatcgtggtgtcacgctcgtcgtttggtatggcttcattcagctccggttcccaacgatcaaggcgagttacatgatcccccatgttgtgcaaaaaagcggttagctccttcggtcctccgatcgttgtcagaagtaagttggccgcagtgttatcactcatggttatggcagcactgcataattctcttactgtcatgccatccgtaagatgcttttctgtgactggtgagtactcaaccaagtcattctgagaatagtgtatgcggcgaccgagttgctcttgcccggcgtcaatacgggataataccgcgccacatagcagaactttaaaagtgctcatcattggaaaacgttcttcggggcgaaaactctcaaggatcttaccgctgttgagatccagttcgatgtaacccactcgtgcacccaactgatcttcagcatcttttactttcaccagcgtttctgggtgagcaaaaacaggaaggcaaaatgccgcaaaaaagggaataagggcgacacggaaatgttgaatactcatactcttcctttttcaatattattgaagcatttatcagggttattgtctcatgagcggatacatatttgaatgtatttagaaaaataaacaaataggggttccgcgcacatttccccgaaaagtgccacctgacgtcgacggatcgggagatctcccgatcccctatggtgcactctcagtacaatctgctctgatgccgcatagttaagccagtatctgctccctgcttgtgtgttggaggtcgctgagtagtgcgcgagcaaaatttaagctacaacaaggcaaggcttgaccgacaattgcatgaagaatctgcttagggttaggcgttttgcgctgcttcgcgatgtacgggccagatata

>TetO-eGFP-FLAG-NLS-RBM20-WT

cgcgttgacattgattattgactagttattaatagtaatcaattacggggtcattagttcatagcccatatatggagttccgcgttacataacttacggtaaatggcccgcctggctgaccgcccaacgacccccgcccattgacgtcaataatgacgtatgttcccatagtaacgccaatagggactttccattgacgtcaatgggtggagtatttacggtaaactgcccacttggcagtacatcaagtgtatcatatgccaagtacgccccctattgacgtcaatgacggtaaatggcccgcctggcattatgcccagtacatgaccttatgggactttcctacttggcagtacatctacgtattagtcatcgctattaccatggtgatgcggttttggcagtacatcaatgggcgtggatagcggtttgactcacggggatttccaagtctccaccccattgacgtcaatgggagtttgttttggcaccaaaatcaacgggactttccaaaatgtcgtaacaactccgccccattgacgcaaatgggcggtaggcgtgtacggtgggaggtctatataagcagcgcgttttgcctgtactgggtctctctggttagaccagatctgagcctgggagctctctggctaactagggaacccactgcttaagcctcaataaagcttgccttgagtgcttcaagtagtgtgtgcccgtctgttgtgtgactctggtaactagagatccctcagacccttttagtcagtgtggaaaatctctagcagtggcgcccgaacagggacttgaaagcgaaagggaaaccagaggagctctctcgacgcaggactcggcttgctgaagcgcgcacggcaagaggcgaggggcggcgactggtgagtacgccaaaaattttgactagcggaggctagaaggagagagatgggtgcgagagcgtcagtattaagcgggggagaattagatcgcgatgggaaaaaattcggttaaggccagggggaaagaaaaaatataaattaaaacatatagtatgggcaagcagggagctagaacgattcgcagttaatcctggcctgttagaaacatcagaaggctgtagacaaatactgggacagctacaaccatcccttcagacaggatcagaagaacttagatcattatataatacagtagcaaccctctattgtgtgcatcaaaggatagagataaaagacaccaaggaagctttagacaagatagaggaagagcaaaacaaaagtaagaccaccgcacagcaagcggccgctgatcttcagacctggaggaggagatatgagggacaattggagaagtgaattatataaatataaagtagtaaaaattgaaccattaggagtagcacccaccaaggcaaagagaagagtggtgcagagagaaaaaagagcagtgggaataggagctttgttccttgggttcttgggagcagcaggaagcactatgggcgcagcgtcaatgacgctgacggtacaggccagacaattattgtctggtatagtgcagcagcagaacaatttgctgagggctattgaggcgcaacagcatctgttgcaactcacagtctggggcatcaagcagctccaggcaagaatcctggctgtggaaagatacctaaaggatcaacagctcctggggatttggggttgctctggaaaactcatttgcaccactgctgtgccttggaatgctagttggagtaataaatctctggaacagatttggaatcacacgacctggatggagtgggacagagaaattaacaattacacaagcttaatacactccttaattgaagaatcgcaaaaccagcaagaaaagaatgaacaagaattattggaattagataaatgggcaagtttgtggaattggtttaacataacaaattggctgtggtatataaaattattcataatgatagtaggaggcttggtaggtttaagaatagtttttgctgtactttctatagtgaatagagttaggcagggatattcaccattatcgtttcagacccacctcccaaccccgaggggacccgacaggcccgaaggaatagaagaagaaggtggagagagagacagagacagatccattcgattagtgaacggatcggcactgcgtgcgccaattctgcagacaaatggcagtattcatccacaattttaaaagaaaaggggggattggggggtacagtgcaggggaaagaatagtagacataatagcaacagacatacaaactaaagaattacaaaaacaaattacaaaaattcaaaattttcgggtttattacagggacagcagagatccagtttggttagatctcgagtttaccactccctatcagtgatagagaaaagtgaaagtcgagtttaccactccctatcagtgatagagaaaagtgaaagtcgagtttaccactccctatcagtgatagagaaaagtgaaagtcgagtttaccactccctatcagtgatagagaaaagtgaaagtcgagtttaccactccctatcagtgatagagaaaagtgaaagtcgagtttaccactccctatcagtgatagagaaaagtgaaagtcgagtttaccactccctatcagtgatagagaaaagtgaaagtcgagctcggtacccgggtcgagtaggcgtgtacggtgggaggcctatataagcagagctcgtttagtgaaccgtcagatcgcctggagacgccatccacgctgttttgacctccatagaagacaccgggaccgatccagcctccgcggccccgaattcgccaccatggtgagcaagggcgaggagctgttcaccggggtggtgcccatcctggtcgagctggacggcgacgtaaacggccacaagttcagcgtgtccggcgagggcgagggcgatgccacctacggcaagctgaccctgaagttcatctgcaccaccggcaagctgcccgtgccctggcccaccctcgtgaccaccctgacctacggcgtgcagtgcttcagccgctaccccgaccacatgaagcagcacgacttcttcaagtccgccatgcccgaaggctacgtccaggagcgcaccatcttcttcaaggacgacggcaactacaagacccgcgccgaggtgaagttcgagggcgacaccctggtgaaccgcatcgagctgaagggcatcgacttcaaggaggacggcaacatcctggggcacaagctggagtacaactacaacagccacaacgtctatatcatggccgacaagcagaagaacggcatcaaggtgaacttcaagatccgccacaacatcgaggacggcagcgtgcagctcgccgaccactaccagcagaacacccccatcggcgacggccccgtgctgctgcccgacaaccactacctgagcacccagtccgccctgagcaaagaccccaacgagaagcgcgatcacatggtcctgctggagttcgtgaccgccgccgggatcactctcggcatggacgagctgtacaagggtggttctggtccaaaaaagaagagaaaggtagactacaaggacgacgatgacaaggtgctggcagcagccatgagccaggacgcggaccccagcggtccggagcagccggacagagttgcctgcagtgtgcctggtgcccgggcgtccccggcaccctccggcccgcgagggatgcagcagccgccgccgccgccccagccaccgcccccgccccaagccggcctaccccagatcatccaaaatgccgccaagctcctggacaagaacccattctcggtcagtaacccgaaccctctgcttccttcacctgccagtctccagctggctcaactgcaggcccagctcaccctccaccggctgaagctggcacagacagctgtcaccaacaacactgcagccgccacagtcctgaaccaagtcctctccaaagtggccatgtcccagcctctcttcaatcaactgaggcatccgtctgtgatcactggcccccacggccatgctggggttccccaacatgctgcagccatacccagcacgcgcttcccatcaaatgcaattgccttttcaccccccagccagacacgaggccccggaccctccatgaaccttcccaaccagccacccagtgccatggtgatgcatcctttcactggggtaatgcctcagacccctggccagccagcagtcatcttgggcattggcaagactgggcctgctccagctacagcaggattctatgagtatggcaaagccagctctggccagacatatggccctgaaacagatggtcagcctggcttcctgccatcctcggcctcaacctcgggcagtgtgacctatgaagggcactacagccacacagggcaggatggtcaagctgccttttccaaagatttttacggacccaactcccaaggttcacatgtggccagcggatttccagctgagcaggctgggggcctgaaaagtgaggtcgggccactgctgcagggcacaaacagccaatgggagagcccccatggattctcgggccaaagcaagcctgatctcacagcaggtcccatgtggcctccaccccacaaccagccctatgagctgtacgaccccgaggaaccaacctcagacaggacacctccttccttcgggggtcggcttaacaacagcaaacagggttttatcggtgctgggcggagggccaaggaggaccaggcgttgctatctgtgcggcccctgcaggctcatgagctgaacgactttcacggtgtggcccccctccacttgccgcatatctgtagcatctgtgacaagaaggtgtttgatttgaaggactgggagctgcatgtgaaaggcaaactccatgcacaaaagtgcctggtcttctctgaaaatgctggcatccggtgtatacttggttcggcagagggaacattgtgtgcttctcccaacagcacagctgtttataaccctgctgggaatgaagattatgcctcaaatcttggaacatcatacgtgcccattccagcaaggtcattcactcagtcaagccccacatttcctttggcttctgtggggacaacttttgcacagcggaaaggggctggccgtgtggtgcacatctgcaatctccctgaaggaagctgcactgagaatgacgtcattaacctggggctgccctttggaaaggtcactaattacatcctcatgaagtcgactaatcaggcctttttagagatggcttacacagaagctgcacaggccatggtccagtattatcaagaaaaatctgctgtgatcaatggtgagaagttgctcattcggatgtccaagagatacaaggaattgcagctcaagaaacccgggaaggccgtggctgccatcatccaggacatccattcccagagggagagggacatgttccgggaagcagacagatatggcccagaaaggccgcggtctcgtagtccggtgagccggtcactctccccgaggtcccacactcccagcttcacctcctgcagctcttcccacagccctccgggcccctcccgggctgactggggcaatggccgggactcctgggagcactctccctatgccaggagggaggaagagcgagacccggctccctggagggacaacggagatgacaagagggacaggatggacccctgggcacatgatcgcaaacaccacccccggcaactggacaaggctgagttggacgagcgaccagaaggagggaggccccaccgggagaagtacccgagatctgggtctcccaacctgccccactctgtgtccagctacaaaagccgtgaagacggctactaccggaaagagcccaaagccaagtcggacaagtatctgaagcagcagcaggatgcccccgggaggtccaggaggaaagacgaggccaggctgcgggaaagcagacacccccatccggatgactcaggcaaggaagatgggctggggccaaaggtcactagggcccctgagggcgccaaggccaagcagaatgagaaaaataaaaccaagagaactgatagagaccaagaaggagctgatgatagaaaagaaaacacaatggcagagaatgaggctggaaaagaggaacaggagggcatggaggagagtccacagtctgtgggcagacaggagaaagaagcagagttctctgatccggaaaacacaaggacaaagaaggaacaagattgggagagtgaaagtgaggcagagggggagagctggtatcccactaacatggaggagctggtgacagtggacgaggttggggaagaagaagattttatcgtggaaccagacatcccagagctggaagaaattgtgcccattgaccagaaagacaaaatttgcccagaaacatgtctgtgtgtgacaaccaccttagacttagacctggcccaggatttccccaaggaaggagtcaaggccgtagggaatggggctgcagaaatcagcctcaagtcacccagagaactgccctctgcttccacaagctgtcccagtgacatggacgtcgagatgccggggcttaacctggatgctgagcggaagccagctgaaagtgagacaggcctctccctggaggattcagattgctacgagaaggaggcaaagggagtggagagctcagatgttcatccagcccctacagtccagcaaatgtcttcccctaagccagcagaggagagggcccggcagccaagcccatttgtggatgattgcaagaccagggggacccccgaagatggggcttgtgaaggcagccccctggaggagaaagccagcccccccatcgaaactgacctccaaaaccaagcttgccaagaagtgttgaccccggaaaactccaggtacgtggaaatgaaatctctggaggtgaggtcaccagagtacactgaagtggaactgaaacagcccctttctttgccctcttgggaaccagaggatgtgttcagtgaacttagcattcctctaggggtggagttcgtggttcccaggactggcttttattgcaagctgtgtgggctgttctacacgagcgaggagacagcaaagatgagccactgccgcagcgctgtccactacaggaacttacagaaatatttgtcccagctggccgaggagggcctcaaggagaccgagggggcagatagcccgaggccagaggacagcggaatcgtgccacgcttcgaaaggaaaaagctctgaggatcctctagagaagactattaattaagctagctaattcgatatcaagcttatcgataatcaacctctggattacaaaatttgtgaaagattgactggtattcttaactatgttgctccttttacgctatgtggatacgctgctttaatgcctttgtatcatgctattgcttcccgtatggctttcattttctcctccttgtataaatcctggttgctgtctctttatgaggagttgtggcccgttgtcaggcaacgtggcgtggtgtgcactgtgtttgctgacgcaacccccactggttggggcattgccaccacctgtcagctcctttccgggactttcgctttccccctccctattgccacggcggaactcatcgccgcctgccttgcccgctgctggacaggggctcggctgttgggcactgacaattccgtggtgttgtcggggaaatcatcgtcctttccttggctgctcgcctgtgttgccacctggattctgcgcgggacgtccttctgctacgtcccttcggccctcaatccagcggaccttccttcccgcggcctgctgccggctctgcggcctcttccgcgtcttcgccttcgccctcagacgagtcggatctccctttgggccgcctccccgcatcgataccgtcgacctcgagacctagaaaaacatggagcaatcacaagtagcaatacagcagctaccaatgctgattgtgcctggctagaagcacaagaggaggaggaggtgggttttccagtcacacctcaggtacctttaagaccaatgacttacaaggcagctgtagatcttagccactttttaaaagaaaaggggggactggaagggctaattcactcccaacgaagacaagatatccttgatctgtggatctaccacacacaaggctacttccctgattggcagaactacacaccagggccagggatcagatatccactgacctttggatggtgctacaagctagtaccagttgagcaagagaaggtagaagaagccaatgaaggagagaacacccgcttgttacaccctgtgagcctgcatgggatggatgacccggagagagaagtattagagtggaggtttgacagccgcctagcatttcatcacatggcccgagagctgcatccggactgtactgggtctctctggttagaccagatctgagcctgggagctctctggctaactagggaacccactgcttaagcctcaataaagcttgccttgagtgcttcaagtagtgtgtgcccgtctgttgtgtgactctggtaactagagatccctcagacccttttagtcagtgtggaaaatctctagcagggcccgtttaaacccgctgatcagcctcgactgtgccttctagttgccagccatctgttgtttgcccctcccccgtgccttccttgaccctggaaggtgccactcccactgtcctttcctaataaaatgaggaaattgcatcgcattgtctgagtaggtgtcattctattctggggggtggggtggggcaggacagcaagggggaggattgggaagacaatagcaggcatgctggggatgcggtgggctctatggcttctgaggcggaaagaaccagctggggctctagggggtatccccacgcgccctgtagcggcgcattaagcgcggcgggtgtggtggttacgcgcagcgtgaccgctacacttgccagcgccctagcgcccgctcctttcgctttcttcccttcctttctcgccacgttcgccggctttccccgtcaagctctaaatcgggggctccctttagggttccgatttagtgctttacggcacctcgaccccaaaaaacttgattagggtgatggttcacgtagtgggccatcgccctgatagacggtttttcgccctttgacgttggagtccacgttctttaatagtggactcttgttccaaactggaacaacactcaaccctatctcggtctattcttttgatttataagggattttgccgatttcggcctattggttaaaaaatgagctgatttaacaaaaatttaacgcgaattaattctgtggaatgtgtgtcagttagggtgtggaaagtccccaggctccccagcaggcagaagtatgcaaagcatgcatctcaattagtcagcaaccaggtgtggaaagtccccaggctccccagcaggcagaagtatgcaaagcatgcatctcaattagtcagcaaccatagtcccgcccctaactccgcccatcccgcccctaactccgcccagttccgcccattctccgccccatggctgactaattttttttatttatgcagaggccgaggccgcctctgcctctgagctattccagaagtagtgaggaggcttttttggaggcctaggcttttgcaaaaagctcccgggagcttgtatatccattttcggatctgatcagcacgtgttgacaattaatcatcggcatagtatatcggcatagtataatacgacaaggtgaggaactaaaccatggccaagttgaccagtgccgttccggtgctcaccgcgcgcgacgtcgccggagcggtcgagttctggaccgaccggctcgggttctcccgggacttcgtggaggacgacttcgccggtgtggtccgggacgacgtgaccctgttcatcagcgcggtccaggaccaggtggtgccggacaacaccctggcctgggtgtgggtgcgcggcctggacgagctgtacgccgagtggtcggaggtcgtgtccacgaacttccgggacgcctccgggccggccatgaccgagatcggcgagcagccgtgggggcgggagttcgccctgcgcgacccggccggcaactgcgtgcacttcgtggccgaggagcaggactgacacgtgctacgagatttcgattccaccgccgccttctatgaaaggttgggcttcggaatcgttttccgggacgccggctggatgatcctccagcgcggggatctcatgctggagttcttcgcccaccccaacttgtttattgcagcttataatggttacaaataaagcaatagcatcacaaatttcacaaataaagcatttttttcactgcattctagttgtggtttgtccaaactcatcaatgtatcttatcatgtctgtataccgtcgacctctagctagagcttggcgtaatcatggtcatagctgtttcctgtgtgaaattgttatccgctcacaattccacacaacatacgagccggaagcataaagtgtaaagcctggggtgcctaatgagtgagctaactcacattaattgcgttgcgctcactgcccgctttccagtcgggaaacctgtcgtgccagctgcattaatgaatcggccaacgcgcggggagaggcggtttgcgtattgggcgctcttccgcttcctcgctcactgactcgctgcgctcggtcgttcggctgcggcgagcggtatcagctcactcaaaggcggtaatacggttatccacagaatcaggggataacgcaggaaagaacatgtgagcaaaaggccagcaaaaggccaggaaccgtaaaaaggccgcgttgctggcgtttttccataggctccgcccccctgacgagcatcacaaaaatcgacgctcaagtcagaggtggcgaaacccgacaggactataaagataccaggcgtttccccctggaagctccctcgtgcgctctcctgttccgaccctgccgcttaccggatacctgtccgcctttctcccttcgggaagcgtggcgctttctcatagctcacgctgtaggtatctcagttcggtgtaggtcgttcgctccaagctgggctgtgtgcacgaaccccccgttcagcccgaccgctgcgccttatccggtaactatcgtcttgagtccaacccggtaagacacgacttatcgccactggcagcagccactggtaacaggattagcagagcgaggtatgtaggcggtgctacagagttcttgaagtggtggcctaactacggctacactagaagaacagtatttggtatctgcgctctgctgaagccagttaccttcggaaaaagagttggtagctcttgatccggcaaacaaaccaccgctggtagcggtggtttttttgtttgcaagcagcagattacgcgcagaaaaaaaggatctcaagaagatcctttgatcttttctacggggtctgacgctcagtggaacgaaaactcacgttaagggattttggtcatgagattatcaaaaaggatcttcacctagatccttttaaattaaaaatgaagttttaaatcaatctaaagtatatatgagtaaacttggtctgacagttaccaatgcttaatcagtgaggcacctatctcagcgatctgtctatttcgttcatccatagttgcctgactccccgtcgtgtagataactacgatacgggagggcttaccatctggccccagtgctgcaatgataccgcgagacccacgctcaccggctccagatttatcagcaataaaccagccagccggaagggccgagcgcagaagtggtcctgcaactttatccgcctccatccagtctattaattgttgccgggaagctagagtaagtagttcgccagttaatagtttgcgcaacgttgttgccattgctacaggcatcgtggtgtcacgctcgtcgtttggtatggcttcattcagctccggttcccaacgatcaaggcgagttacatgatcccccatgttgtgcaaaaaagcggttagctccttcggtcctccgatcgttgtcagaagtaagttggccgcagtgttatcactcatggttatggcagcactgcataattctcttactgtcatgccatccgtaagatgcttttctgtgactggtgagtactcaaccaagtcattctgagaatagtgtatgcggcgaccgagttgctcttgcccggcgtcaatacgggataataccgcgccacatagcagaactttaaaagtgctcatcattggaaaacgttcttcggggcgaaaactctcaaggatcttaccgctgttgagatccagttcgatgtaacccactcgtgcacccaactgatcttcagcatcttttactttcaccagcgtttctgggtgagcaaaaacaggaaggcaaaatgccgcaaaaaagggaataagggcgacacggaaatgttgaatactcatactcttcctttttcaatattattgaagcatttatcagggttattgtctcatgagcggatacatatttgaatgtatttagaaaaataaacaaataggggttccgcgcacatttccccgaaaagtgccacctgacgtcgacggatcgggagatctcccgatcccctatggtgcactctcagtacaatctgctctgatgccgcatagttaagccagtatctgctccctgcttgtgtgttggaggtcgctgagtagtgcgcgagcaaaatttaagctacaacaaggcaaggcttgaccgacaattgcatgaagaatctgcttagggttaggcgttttgcgctgcttcgcgatgtacgggccagatata

>pEFa-eGFP-FLAG-RBM20-WT

cgcgttgacattgattattgactagttattaatagtaatcaattacggggtcattagttcatagcccatatatggagttccgcgttacataacttacggtaaatggcccgcctggctgaccgcccaacgacccccgcccattgacgtcaataatgacgtatgttcccatagtaacgccaatagggactttccattgacgtcaatgggtggagtatttacggtaaactgcccacttggcagtacatcaagtgtatcatatgccaagtacgccccctattgacgtcaatgacggtaaatggcccgcctggcattatgcccagtacatgaccttatgggactttcctacttggcagtacatctacgtattagtcatcgctattaccatggtgatgcggttttggcagtacatcaatgggcgtggatagcggtttgactcacggggatttccaagtctccaccccattgacgtcaatgggagtttgttttggcaccaaaatcaacgggactttccaaaatgtcgtaacaactccgccccattgacgcaaatgggcggtaggcgtgtacggtgggaggtctatataagcagcgcgttttgcctgtactgggtctctctggttagaccagatctgagcctgggagctctctggctaactagggaacccactgcttaagcctcaataaagcttgccttgagtgcttcaagtagtgtgtgcccgtctgttgtgtgactctggtaactagagatccctcagacccttttagtcagtgtggaaaatctctagcagtggcgcccgaacagggacttgaaagcgaaagggaaaccagaggagctctctcgacgcaggactcggcttgctgaagcgcgcacggcaagaggcgaggggcggcgactggtgagtacgccaaaaattttgactagcggaggctagaaggagagagatgggtgcgagagcgtcagtattaagcgggggagaattagatcgcgatgggaaaaaattcggttaaggccagggggaaagaaaaaatataaattaaaacatatagtatgggcaagcagggagctagaacgattcgcagttaatcctggcctgttagaaacatcagaaggctgtagacaaatactgggacagctacaaccatcccttcagacaggatcagaagaacttagatcattatataatacagtagcaaccctctattgtgtgcatcaaaggatagagataaaagacaccaaggaagctttagacaagatagaggaagagcaaaacaaaagtaagaccaccgcacagcaagcggccgctgatcttcagacctggaggaggagatatgagggacaattggagaagtgaattatataaatataaagtagtaaaaattgaaccattaggagtagcacccaccaaggcaaagagaagagtggtgcagagagaaaaaagagcagtgggaataggagctttgttccttgggttcttgggagcagcaggaagcactatgggcgcagcgtcaatgacgctgacggtacaggccagacaattattgtctggtatagtgcagcagcagaacaatttgctgagggctattgaggcgcaacagcatctgttgcaactcacagtctggggcatcaagcagctccaggcaagaatcctggctgtggaaagatacctaaaggatcaacagctcctggggatttggggttgctctggaaaactcatttgcaccactgctgtgccttggaatgctagttggagtaataaatctctggaacagatttggaatcacacgacctggatggagtgggacagagaaattaacaattacacaagcttaatacactccttaattgaagaatcgcaaaaccagcaagaaaagaatgaacaagaattattggaattagataaatgggcaagtttgtggaattggtttaacataacaaattggctgtggtatataaaattattcataatgatagtaggaggcttggtaggtttaagaatagtttttgctgtactttctatagtgaatagagttaggcagggatattcaccattatcgtttcagacccacctcccaaccccgaggggacccgacaggcccgaaggaatagaagaagaaggtggagagagagacagagacagatccattcgattagtgaacggatcggcactgcgtgcgccaattctgcagacaaatggcagtattcatccacaattttaaaagaaaaggggggattggggggtacagtgcaggggaaagaatagtagacataatagcaacagacatacaaactaaagaattacaaaaacaaattacaaaaattcaaaattttcgggtttattacagggacagcagagatccactttggcgccggctcgagtggctccggtgcccgtcagtgggcagagcgcacatcgcccacagtccccgagaagttggggggaggggtcggcaattgaaccggtgcctagagaaggtggcgcggggtaaactgggaaagtgatgtcgtgtactggctccgcctttttcccgagggtgggggagaaccgtatataagtgcagtagtcgccgtgaacgttctttttcgcaacgggtttgccgccagaacacaggtgtcgtgacgcgggatccgccaccatggtgagcaagggcgaggagctgttcaccggggtggtgcccatcctggtcgagctggacggcgacgtaaacggccacaagttcagcgtgtccggcgagggcgagggcgatgccacctacggcaagctgaccctgaagttcatctgcaccaccggcaagctgcccgtgccctggcccaccctcgtgaccaccctgacctacggcgtgcagtgcttcagccgctaccccgaccacatgaagcagcacgacttcttcaagtccgccatgcccgaaggctacgtccaggagcgcaccatcttcttcaaggacgacggcaactacaagacccgcgccgaggtgaagttcgagggcgacaccctggtgaaccgcatcgagctgaagggcatcgacttcaaggaggacggcaacatcctggggcacaagctggagtacaactacaacagccacaacgtctatatcatggccgacaagcagaagaacggcatcaaggtgaacttcaagatccgccacaacatcgaggacggcagcgtgcagctcgccgaccactaccagcagaacacccccatcggcgacggccccgtgctgctgcccgacaaccactacctgagcacccagtccgccctgagcaaagaccccaacgagaagcgcgatcacatggtcctgctggagttcgtgaccgccgccgggatcactctcggcatggacgagctgtacaagggtggttctggtgactacaaggacgacgatgacaaggtgctggcagcagccatgagccaggacgcggaccccagcggtccggagcagccggacagagttgcctgcagtgtgcctggtgcccgggcgtccccggcaccctccggcccgcgagggatgcagcagccgccgccgccgccccagccaccgcccccgccccaagccggcctaccccagatcatccaaaatgccgccaagctcctggacaagaacccattctcggtcagtaacccgaaccctctgcttccttcacctgccagtctccagctggctcaactgcaggcccagctcaccctccaccggctgaagctggcacagacagctgtcaccaacaacactgcagccgccacagtcctgaaccaagtcctctccaaagtggccatgtcccagcctctcttcaatcaactgaggcatccgtctgtgatcactggcccccacggccatgctggggttccccaacatgctgcagccatacccagcacgcgcttcccatcaaatgcaattgccttttcaccccccagccagacacgaggccccggaccctccatgaaccttcccaaccagccacccagtgccatggtgatgcatcctttcactggggtaatgcctcagacccctggccagccagcagtcatcttgggcattggcaagactgggcctgctccagctacagcaggattctatgagtatggcaaagccagctctggccagacatatggccctgaaacagatggtcagcctggcttcctgccatcctcggcctcaacctcgggcagtgtgacctatgaagggcactacagccacacagggcaggatggtcaagctgccttttccaaagatttttacggacccaactcccaaggttcacatgtggccagcggatttccagctgagcaggctgggggcctgaaaagtgaggtcgggccactgctgcagggcacaaacagccaatgggagagcccccatggattctcgggccaaagcaagcctgatctcacagcaggtcccatgtggcctccaccccacaaccagccctatgagctgtacgaccccgaggaaccaacctcagacaggacacctccttccttcgggggtcggcttaacaacagcaaacagggttttatcggtgctgggcggagggccaaggaggaccaggcgttgctatctgtgcggcccctgcaggctcatgagctgaacgactttcacggtgtggcccccctccacttgccgcatatctgtagcatctgtgacaagaaggtgtttgatttgaaggactgggagctgcatgtgaaaggcaaactccatgcacaaaagtgcctggtcttctctgaaaatgctggcatccggtgtatacttggttcggcagagggaacattgtgtgcttctcccaacagcacagctgtttataaccctgctgggaatgaagattatgcctcaaatcttggaacatcatacgtgcccattccagcaaggtcattcactcagtcaagccccacatttcctttggcttctgtggggacaacttttgcacagcggaaaggggctggccgtgtggtgcacatctgcaatctccctgaaggaagctgcactgagaatgacgtcattaacctggggctgccctttggaaaggtcactaattacatcctcatgaagtcgactaatcaggcctttttagagatggcttacacagaagctgcacaggccatggtccagtattatcaagaaaaatctgctgtgatcaatggtgagaagttgctcattcggatgtccaagagatacaaggaattgcagctcaagaaacccgggaaggccgtggctgccatcatccaggacatccattcccagagggagagggacatgttccgggaagcagacagatatggcccagaaaggccgcggtctcgtagtccggtgagccggtcactctccccgaggtcccacactcccagcttcacctcctgcagctcttcccacagccctccgggcccctcccgggctgactggggcaatggccgggactcctgggagcactctccctatgccaggagggaggaagagcgagacccggctccctggagggacaacggagatgacaagagggacaggatggacccctgggcacatgatcgcaaacaccacccccggcaactggacaaggctgagttggacgagcgaccagaaggagggaggccccaccgggagaagtacccgagatctgggtctcccaacctgccccactctgtgtccagctacaaaagccgtgaagacggctactaccggaaagagcccaaagccaagtcggacaagtatctgaagcagcagcaggatgcccccgggaggtccaggaggaaagacgaggccaggctgcgggaaagcagacacccccatccggatgactcaggcaaggaagatgggctggggccaaaggtcactagggcccctgagggcgccaaggccaagcagaatgagaaaaataaaaccaagagaactgatagagaccaagaaggagctgatgatagaaaagaaaacacaatggcagagaatgaggctggaaaagaggaacaggagggcatggaggagagtccacagtctgtgggcagacaggagaaagaagcagagttctctgatccggaaaacacaaggacaaagaaggaacaagattgggagagtgaaagtgaggcagagggggagagctggtatcccactaacatggaggagctggtgacagtggacgaggttggggaagaagaagattttatcgtggaaccagacatcccagagctggaagaaattgtgcccattgaccagaaagacaaaatttgcccagaaacatgtctgtgtgtgacaaccaccttagacttagacctggcccaggatttccccaaggaaggagtcaaggccgtagggaatggggctgcagaaatcagcctcaagtcacccagagaactgccctctgcttccacaagctgtcccagtgacatggacgtcgagatgccggggcttaacctggatgctgagcggaagccagctgaaagtgagacaggcctctccctggaggattcagattgctacgagaaggaggcaaagggagtggagagctcagatgttcatccagcccctacagtccagcaaatgtcttcccctaagccagcagaggagagggcccggcagccaagcccatttgtggatgattgcaagaccagggggacccccgaagatggggcttgtgaaggcagccccctggaggagaaagccagcccccccatcgaaactgacctccaaaaccaagcttgccaagaagtgttgaccccggaaaactccaggtacgtggaaatgaaatctctggaggtgaggtcaccagagtacactgaagtggaactgaaacagcccctttctttgccctcttgggaaccagaggatgtgttcagtgaacttagcattcctctaggggtggagttcgtggttcccaggactggcttttattgcaagctgtgtgggctgttctacacgagcgaggagacagcaaagatgagccactgccgcagcgctgtccactacaggaacttacagaaatatttgtcccagctggccgaggagggcctcaaggagaccgagggggcagatagcccgaggccagaggacagcggaatcgtgccacgcttcgaaaggaaaaagctctgaggatcctctagagaagactattaattaagctagctaattcgatatcaagcttatcgataatcaacctctggattacaaaatttgtgaaagattgactggtattcttaactatgttgctccttttacgctatgtggatacgctgctttaatgcctttgtatcatgctattgcttcccgtatggctttcattttctcctccttgtataaatcctggttgctgtctctttatgaggagttgtggcccgttgtcaggcaacgtggcgtggtgtgcactgtgtttgctgacgcaacccccactggttggggcattgccaccacctgtcagctcctttccgggactttcgctttccccctccctattgccacggcggaactcatcgccgcctgccttgcccgctgctggacaggggctcggctgttgggcactgacaattccgtggtgttgtcggggaaatcatcgtcctttccttggctgctcgcctgtgttgccacctggattctgcgcgggacgtccttctgctacgtcccttcggccctcaatccagcggaccttccttcccgcggcctgctgccggctctgcggcctcttccgcgtcttcgccttcgccctcagacgagtcggatctccctttgggccgcctccccgcatcgataccgtcgacctcgagacctagaaaaacatggagcaatcacaagtagcaatacagcagctaccaatgctgattgtgcctggctagaagcacaagaggaggaggaggtgggttttccagtcacacctcaggtacctttaagaccaatgacttacaaggcagctgtagatcttagccactttttaaaagaaaaggggggactggaagggctaattcactcccaacgaagacaagatatccttgatctgtggatctaccacacacaaggctacttccctgattggcagaactacacaccagggccagggatcagatatccactgacctttggatggtgctacaagctagtaccagttgagcaagagaaggtagaagaagccaatgaaggagagaacacccgcttgttacaccctgtgagcctgcatgggatggatgacccggagagagaagtattagagtggaggtttgacagccgcctagcatttcatcacatggcccgagagctgcatccggactgtactgggtctctctggttagaccagatctgagcctgggagctctctggctaactagggaacccactgcttaagcctcaataaagcttgccttgagtgcttcaagtagtgtgtgcccgtctgttgtgtgactctggtaactagagatccctcagacccttttagtcagtgtggaaaatctctagcagggcccgtttaaacccgctgatcagcctcgactgtgccttctagttgccagccatctgttgtttgcccctcccccgtgccttccttgaccctggaaggtgccactcccactgtcctttcctaataaaatgaggaaattgcatcgcattgtctgagtaggtgtcattctattctggggggtggggtggggcaggacagcaagggggaggattgggaagacaatagcaggcatgctggggatgcggtgggctctatggcttctgaggcggaaagaaccagctggggctctagggggtatccccacgcgccctgtagcggcgcattaagcgcggcgggtgtggtggttacgcgcagcgtgaccgctacacttgccagcgccctagcgcccgctcctttcgctttcttcccttcctttctcgccacgttcgccggctttccccgtcaagctctaaatcgggggctccctttagggttccgatttagtgctttacggcacctcgaccccaaaaaacttgattagggtgatggttcacgtagtgggccatcgccctgatagacggtttttcgccctttgacgttggagtccacgttctttaatagtggactcttgttccaaactggaacaacactcaaccctatctcggtctattcttttgatttataagggattttgccgatttcggcctattggttaaaaaatgagctgatttaacaaaaatttaacgcgaattaattctgtggaatgtgtgtcagttagggtgtggaaagtccccaggctccccagcaggcagaagtatgcaaagcatgcatctcaattagtcagcaaccaggtgtggaaagtccccaggctccccagcaggcagaagtatgcaaagcatgcatctcaattagtcagcaaccatagtcccgcccctaactccgcccatcccgcccctaactccgcccagttccgcccattctccgccccatggctgactaattttttttatttatgcagaggccgaggccgcctctgcctctgagctattccagaagtagtgaggaggcttttttggaggcctaggcttttgcaaaaagctcccgggagcttgtatatccattttcggatctgatcagcacgtgttgacaattaatcatcggcatagtatatcggcatagtataatacgacaaggtgaggaactaaaccatggccaagttgaccagtgccgttccggtgctcaccgcgcgcgacgtcgccggagcggtcgagttctggaccgaccggctcgggttctcccgggacttcgtggaggacgacttcgccggtgtggtccgggacgacgtgaccctgttcatcagcgcggtccaggaccaggtggtgccggacaacaccctggcctgggtgtgggtgcgcggcctggacgagctgtacgccgagtggtcggaggtcgtgtccacgaacttccgggacgcctccgggccggccatgaccgagatcggcgagcagccgtgggggcgggagttcgccctgcgcgacccggccggcaactgcgtgcacttcgtggccgaggagcaggactgacacgtgctacgagatttcgattccaccgccgccttctatgaaaggttgggcttcggaatcgttttccgggacgccggctggatgatcctccagcgcggggatctcatgctggagttcttcgcccaccccaacttgtttattgcagcttataatggttacaaataaagcaatagcatcacaaatttcacaaataaagcatttttttcactgcattctagttgtggtttgtccaaactcatcaatgtatcttatcatgtctgtataccgtcgacctctagctagagcttggcgtaatcatggtcatagctgtttcctgtgtgaaattgttatccgctcacaattccacacaacatacgagccggaagcataaagtgtaaagcctggggtgcctaatgagtgagctaactcacattaattgcgttgcgctcactgcccgctttccagtcgggaaacctgtcgtgccagctgcattaatgaatcggccaacgcgcggggagaggcggtttgcgtattgggcgctcttccgcttcctcgctcactgactcgctgcgctcggtcgttcggctgcggcgagcggtatcagctcactcaaaggcggtaatacggttatccacagaatcaggggataacgcaggaaagaacatgtgagcaaaaggccagcaaaaggccaggaaccgtaaaaaggccgcgttgctggcgtttttccataggctccgcccccctgacgagcatcacaaaaatcgacgctcaagtcagaggtggcgaaacccgacaggactataaagataccaggcgtttccccctggaagctccctcgtgcgctctcctgttccgaccctgccgcttaccggatacctgtccgcctttctcccttcgggaagcgtggcgctttctcatagctcacgctgtaggtatctcagttcggtgtaggtcgttcgctccaagctgggctgtgtgcacgaaccccccgttcagcccgaccgctgcgccttatccggtaactatcgtcttgagtccaacccggtaagacacgacttatcgccactggcagcagccactggtaacaggattagcagagcgaggtatgtaggcggtgctacagagttcttgaagtggtggcctaactacggctacactagaagaacagtatttggtatctgcgctctgctgaagccagttaccttcggaaaaagagttggtagctcttgatccggcaaacaaaccaccgctggtagcggtggtttttttgtttgcaagcagcagattacgcgcagaaaaaaaggatctcaagaagatcctttgatcttttctacggggtctgacgctcagtggaacgaaaactcacgttaagggattttggtcatgagattatcaaaaaggatcttcacctagatccttttaaattaaaaatgaagttttaaatcaatctaaagtatatatgagtaaacttggtctgacagttaccaatgcttaatcagtgaggcacctatctcagcgatctgtctatttcgttcatccatagttgcctgactccccgtcgtgtagataactacgatacgggagggcttaccatctggccccagtgctgcaatgataccgcgagacccacgctcaccggctccagatttatcagcaataaaccagccagccggaagggccgagcgcagaagtggtcctgcaactttatccgcctccatccagtctattaattgttgccgggaagctagagtaagtagttcgccagttaatagtttgcgcaacgttgttgccattgctacaggcatcgtggtgtcacgctcgtcgtttggtatggcttcattcagctccggttcccaacgatcaaggcgagttacatgatcccccatgttgtgcaaaaaagcggttagctccttcggtcctccgatcgttgtcagaagtaagttggccgcagtgttatcactcatggttatggcagcactgcataattctcttactgtcatgccatccgtaagatgcttttctgtgactggtgagtactcaaccaagtcattctgagaatagtgtatgcggcgaccgagttgctcttgcccggcgtcaatacgggataataccgcgccacatagcagaactttaaaagtgctcatcattggaaaacgttcttcggggcgaaaactctcaaggatcttaccgctgttgagatccagttcgatgtaacccactcgtgcacccaactgatcttcagcatcttttactttcaccagcgtttctgggtgagcaaaaacaggaaggcaaaatgccgcaaaaaagggaataagggcgacacggaaatgttgaatactcatactcttcctttttcaatattattgaagcatttatcagggttattgtctcatgagcggatacatatttgaatgtatttagaaaaataaacaaataggggttccgcgcacatttccccgaaaagtgccacctgacgtcgacggatcgggagatctcccgatcccctatggtgcactctcagtacaatctgctctgatgccgcatagttaagccagtatctgctccctgcttgtgtgttggaggtcgctgagtagtgcgcgagcaaaatttaagctacaacaaggcaaggcttgaccgacaattgcatgaagaatctgcttagggttaggcgttttgcgctgcttcgcgatgtacgggccagatata

>AAV9-pCMV-Tnpo3

cctgcaggcagctgcgcgctcgctcgctcactgaggccgcccgggcaaagcccgggcgtcgggcgacctttggtcgcccggcctcagtgagcgagcgagcgcgcagagagggagtggccaactccatcactaggggttcctgcggccgcgtcgaccgtacccgttacataacttacggtaaatggcccgcctggctgaccgcccaacgacccccgcccattgacgtcaatagtaacgccaatagggactttccattgacgtcaatgggtggagtatttacggtaaactgcccacttggcagtacatcaagtgtatcatatgccaagtacgccccctattgacgtcaatgacggtaaatggcccgcctggcattgtgcccagtacatgaccttatgggactttcctacttggcagtacatctacgtattagtcatcgctattaccatggtcgaggtgagccccacgttctgcttcactctccccatctcccccccctccccacccccaattttgtatttatttattttttaattattttgtgcagcgatgggggcggggggggggggggggcgcgcgccaggcggggcggggcggggcgaggggcggggcggggcgaggcggagaggtgcggcggcagccaatcagagcggcgcgctccgaaagtttccttttatggcgaggcggcggcggcggcggccctataaaaagcgaagcgcgcggcgggcgggagtcgctgcgacgctgccttcgccccgtgccccgctccgccgccgcctcgcgccgcccgccccggctctgactgaccgcgttactcccacaggtgagcgggcgggacggcccttctcctccgggctgtaattagctgagcaagaggtaagggtttaagggatggttggttggtggggtattaatgtttaattacctggagcacctgcctgaaatcactttttttcaggttggaggtaccatggagggagccaagccaaccttgcagctcgtgtaccaggcggtgcaagcgctttaccacgacccggatcccagcggaaaggagcgcgcctcgttttggcttggggagctgcagcgttcggttcacgcttgggagatttctgatcaattgttacagatccgacaggatgtggaatcatgctatttcgctgcccagaccatgaaaatgaagattcagacctcattttatgagctccccacagactctcatgcttctttaagggactcattgctaactcacattcagaacttgaaagacttgtcgcctgtcattgtaacacagctggctttagcaatagcagacctcgccctacagatgccttcttggaaaggatgcgtacaaacattggtagaaaaatatagcaatgacgtaacttctttaccttttttgctggaaatccttacagtgttacccgaagaagtacatagtcgttccttgcggattggggccaacaggcggacagaaattatagaagacttagccttctactctagtaccgtggtatctttattgatgacatgtgtagagaaagcaggaaccgatgagaagatgcttatgaaggtcttccgatgtttgggaagttggtttaacttgggggttttggacagtaacttcatggctaataataagttattagcactcctttttgaggtcttgcaacaagataagacctcatctaacctacacgaagctgcttcagactgtgtatgctcagctctctatgctattgaaaatgtagagactaacttgccattggccatgcagctttttcagggagtcctgacattggagactgcatatcatatggctgtggcacgggaagatttggacaaagttctgaattactgccgaattttcaccgaactatgtgaaactttcctggaaaaaattgtctgtactccaggccaaggtctgggagatcttcgaactttggaattgcttcttatctgtgcaggccaccctcagtatgaggtagtagaaatttcctttaacttttggtaccgactaggagaacatttatacaaaaccaatgatgaagttattcacagcatcttcaaagcgtacattcagaggctgcttcatgccttggctcgacactgccagttggaaccagaccatgagggagttcctgaagagaccgatgactttggggagtttcgaatgagagtgtcagacctggtgaaggacttgatcttcctgattggatctatggagtgttttgctcagttgtattctactctgaaggaaggcaacccaccctgggaggtgacagaagcggttctctttatcatggctgctatagcaaagagtgtggatccggagaacaaccctacacttgtggaggtactagagggagttgtccatctcccagagaccgtgcatacagctgtgcggtacactagcattgagttggttggtgagatgagtgaagtggtggaccggaatccccagttcctggaccctgtattgggctatttgatgaaaggcctgtgtgaaaagcctctggcttctgctgcagccaaagccattcataacatttgttccgtttgtcgcgatcatatggctcagcactttaatggactcctagaaattgcccactcccttgattctttcatgttgtctcccgaagctgctgtgggtttgctaaaaggaacagctcttgtcctagcaagactacctttggataagattacagaatgtctaagtgagttatgttctgttcaggttatggcattgaaaaagctgttgtctcaggagcccagcaatggcatttcttcagatcccactgtcttcttagatcgcctggcagtaatatttagacacaccaatcctattgtagaaaatggacagactcatccatgccaaaaagtcatccaggaaatatggccagttttgtctgagactctaaataagcaccgagccgacaatcgaattgtagagcgttgttgtagatgcctgcgctttgcggttcgttgtgtaggcaaaggatctgcagcactgctacagccactagttacacagatggtgaatgtgtaccatgtacatcagcattcctgtttcctctaccttggcagtatccttgtggatgagtacggcatggaagaaggctgtcggcaggggttattagacatgctccaggcgctatgcattcccacctttcagctcctagaacagcagaatggcctccaaaatcaccctgacactgtagatgacctattcaggttagccacccgatttattcagcgaagccctgtcaccttgctgaggagccaggttgtcatcccaatcttacagtgggccattgcatcaaccactttggaccaccgagatgccaattccagtgtcatgagatttctgcgagacctcatccacacaggagtagccaatgatcatgaagaagattttgaattgcggaaggaactaattggacaggtgatgagccagcttggccagcaacttgtcagccagctgctccacacatgctgcttttgtcttcccccctacaccctacccgacgtggctgaagtgctctgggagatcatgcaggttgacagaccgactttctgtcggtggctagagaattccttgaaaggtttgccaaaagagaccacagtgggagctgtcacagtgacacataaacaacttacagatttccacaagcaagtcactagtgccgaggaatgtaagcaagtttgctgggccttgagagacttcaccaggttgtttcgatagggatccgatctgataatcaacctctggattacaaaatttgtgaaagattgactggtattcttaactatgttgctccttttacgctatgtggatacgctgctttaatgcctttgtatcatgctattgcttcccgtatggctttcattttctcctccttgtataaatcctggttagttcttgccacggcggaactcatcgccgcctgccttgcccgctgctggacaggggctcggctgttgggcactgacaattccgtggtgcgactgtgccttctagttgccagccatctgttgtttgcccctcccccgtgccttccttgaccctggaaggtgccactcccactgtcctttcctaataaaatgaggaaattgcatcgcattgtctgagtaggtgtcattctattctggggggtggggtggggcaggacagcaagggggaggattgggaagacaatagcaggcatgctggggatgcggtgggctctatgggcggccgcaggaacccctagtgatggagttggccactccctctctgcgcgctcgctcgctcactgaggccgggcgaccaaaggtcgcccgacgcccgggctttgcccgggcggcctcagtgagcgagcgagcgcgcagctgcctgcaggggcgcctgatgcggtattttctccttacgcatctgtgcggtatttcacaccgcatacgtcaaagcaaccatagtacgcgccctgtagcggcgcattaagcgcggcgggtgtggtggttacgcgcagcgtgaccgctacacttgccagcgccctagcgcccgctcctttcgctttcttcccttcctttctcgccacgttcgccggctttccccgtcaagctctaaatcgggggctccctttagggttccgatttagtgctttacggcacctcgaccccaaaaaacttgatttgggtgatggttcacgtagtgggccatcgccctgatagacggtttttcgccctttgacgttggagtccacgttctttaatagtggactcttgttccaaactggaacaacactcaaccctatctcgggctattcttttgatttataagggattttgccgatttcggcctattggttaaaaaatgagctgatttaacaaaaatttaacgcgaattttaacaaaatattaacgtttacaattttatggtgcactctcagtacaatctgctctgatgccgcatagttaagccagccccgacacccgccaacacccgctgacgcgccctgacgggcttgtctgctcccggcatccgcttacagacaagctgtgaccgtctccgggagctgcatgtgtcagaggttttcaccgtcatcaccgaaacgcgcgagacgaaagggcctcgtgatacgcctatttttataggttaatgtcatgataataatggtttcttagacgtcaggtggcacttttcggggaaatgtgcgcggaacccctatttgtttatttttctaaatacattcaaatatgtatccgctcatgagacaataaccctgataaatgcttcaataatattgaaaaaggaagagtatgagtattcaacatttccgtgtcgcccttattcccttttttgcggcattttgccttcctgtttttgctcacccagaaacgctggtgaaagtaaaagatgctgaagatcagttgggtgcacgagtgggttacatcgaactggatctcaacagcggtaagatccttgagagttttcgccccgaagaacgttttccaatgatgagcacttttaaagttctgctatgtggcgcggtattatcccgtattgacgccgggcaagagcaactcggtcgccgcatacactattctcagaatgacttggttgagtactcaccagtcacagaaaagcatcttacggatggcatgacagtaagagaattatgcagtgctgccataaccatgagtgataacactgcggccaacttacttctgacaacgatcggaggaccgaaggagctaaccgcttttttgcacaacatgggggatcatgtaactcgccttgatcgttgggaaccggagctgaatgaagccataccaaacgacgagcgtgacaccacgatgcctgtagcaatggcaacaacgttgcgcaaactattaactggcgaactacttactctagcttcccggcaacaattaatagactggatggaggcggataaagttgcaggaccacttctgcgctcggcccttccggctggctggtttattgctgataaatctggagccggtgagcgtgggtctcgcggtatcattgcagcactggggccagatggtaagccctcccgtatcgtagttatctacacgacggggagtcaggcaactatggatgaacgaaatagacagatcgctgagataggtgcctcactgattaagcattggtaactgtcagaccaagtttactcatatatactttagattgatttaaaacttcatttttaatttaaaaggatctaggtgaagatcctttttgataatctcatgaccaaaatcccttaacgtgagttttcgttccactgagcgtcagaccccgtagaaaagatcaaaggatcttcttgagatcctttttttctgcgcgtaatctgctgcttgcaaacaaaaaaaccaccgctaccagcggtggtttgtttgccggatcaagagctaccaactctttttccgaaggtaactggcttcagcagagcgcagataccaaatactgtccttctagtgtagccgtagttaggccaccacttcaagaactctgtagcaccgcctacatacctcgctctgctaatcctgttaccagtggctgctgccagtggcgataagtcgtgtcttaccgggttggactcaagacgatagttaccggataaggcgcagcggtcgggctgaacggggggttcgtgcacacagcccagcttggagcgaacgacctacaccgaactgagatacctacagcgtgagctatgagaaagcgccacgcttcccgaagggagaaaggcggacaggtatccggtaagcggcagggtcggaacaggagagcgcacgagggagcttccagggggaaacgcctggtatctttatagtcctgtcgggtttcgccacctctgacttgagcgtcgatttttgtgatgctcgtcaggggggcggagcctatggaaaaacgccagcaacgcggcctttttacggttcctggccttttgctggccttttgctcacatgt

>CROPSeq-guide(F+E)-Puro backbone

aggagctttgttccttgggttcttgggagcagcaggaagcactatgggcgcagcgtcaatgacgctgacggtacaggccagacaattattgtctggtatagtgcagcagcagaacaatttgctgagggctattgaggcgcaacagcatctgttgcaactcacagtctggggcatcaagcagctccaggcaagaatcctggctgtggaaagatacctaaaggatcaacagctcctggggatttggggttgctctggaaaactcatttgcaccactgctgtgccttggaatgctagttggagtaataaatctctggaacagatttggaatcacacgacctggatggagtgggacagagaaattaacaattacacaagcttaatacactccttaattgaagaatcgcaaaaccagcaagaaaagaatgaacaagaattattggaattagataaatgggcaagtttgtggaattggtttaacataacaaattggctgtggtatataaaattattcataatgatagtaggaggcttggtaggtttaagaatagtttttgctgtactttctatagtgaatagagttaggcagggatattcaccattatcgtttcagaatcttgagacaaatggcagtattcatccacaattttaaaagaaaaggggggattggggggtacagtgcaggggaaagaatagtagacataatagcaacagacatacaaactaaagaattacaaaaacaaattacaaaaattcaaaattttcgggtttattacagggacagcagagatccactttggcgccggctcgagggggcccgggtgcaaagatggataaagttttaaacagagaggaatctttgcagctaatggaccttctaggtcttgaaaggagtgggaattggctccggtgcccgtcagtgggcagagcgcacatcgcccacagtccccgagaagttggggggaggggtcggcaattgatccggtgcctagagaaggtggcgcggggtaaactgggaaagtgatgtcgtgtactggctccgcctttttcccgagggtgggggagaaccgtatataagtgcagtagtcgccgtgaacgttctttttcgcaacgggtttgccgccagaacacaggtaagtgccgtgtgtggttcccgcgggcctggcctctttacgggttatggcccttgcgtgccttgaattacttccacctggctgcagtacgtgattcttgatcccgagcttcgggttggaagtgggtgggagagttcgaggccttgcgcttaaggagccccttcgcctcgtgcttgagttgaggcctggcctgggcgctggggccgccgcgtgcgaatctggtggcaccttcgcgcctgtctcgctgctttcgataagtctctagccatttaaaatttttgatgacctgctgcgacgctttttttctggcaagatagtcttgtaaatgcgggccaagatctgcacactggtatttcggtttttggggccgcgggcggcgacggggcccgtgcgtcccagcgcacatgttcggcgaggcggggcctgcgagcgcggccaccgagaatcggacgggggtagtctcaagctggccggcctgctctggtgcctggcctcgcgccgccgtgtatcgccccgccctgggcggcaaggctggcccggtcggcaccagttgcgtgagcggaaagatggccgcttcccggccctgctgcagggagctcaaaatggaggacgcggcgctcgggagagcgggcgggtgagtcacccacacaaaggaaaagggcctttccgtcctcagccgtcgcttcatgtgactccacggagtaccgggcgccgtccaggcacctcgattagttctcgagcttttggagtacgtcgtctttaggttggggggaggggttttatgcgatggagtttccccacactgagtgggtggagactgaagttaggccagcttggcacttgatgtaattctccttggaatttgccctttttgagtttggatcttggttcattctcaagcctcagacagtggttcaaagtttttttcttccatttcaggtgtcgtgacgtacggccaccatgaccgagtacaagcccacggtgcgcctcgccacccgcgacgacgtccccagggccgtacgcaccctcgccgccgcgttcgccgactaccccgccacgcgccacaccgtcgatccggaccgccacatcgagcgggtcaccgagctgcaagaactcttcctcacgcgcgtcgggctcgacatcggcaaggtgtgggtcgcggacgacggcgccgccgtggcggtctggaccacgccggagagcgtcgaagcgggggcggtgttcgccgagatcggcccgcgcatggccgagttgagcggttcccggctggccgcgcagcaacagatggaaggcctcctggcgccgcaccggcccaaggagcccgcgtggttcctggccaccgtcggagtctcgcccgaccaccagggcaagggtctgggcagcgccgtcgtgctccccggagtggaggcggccgagcgcgccggggtgcccgccttcctggagacctccgcgccccgcaacctccccttctacgagcggctcggcttcaccgtcaccgccgacgtcgaggtgcccgaaggaccgcgcacctggtgcatgacccgcaagcccggtgcctgaacgcgttaagtcgacaatcaacctctggattacaaaatttgtgaaagattgactggtattcttaactatgttgctccttttacgctatgtggatacgctgctttaatgcctttgtatcatgctattgcttcccgtatggctttcattttctcctccttgtataaatcctggttgctgtctctttatgaggagttgtggcccgttgtcaggcaacgtggcgtggtgtgcactgtgtttgctgacgcaacccccactggttggggcattgccaccacctgtcagctcctttccgggactttcgctttccccctccctattgccacggcggaactcatcgccgcctgccttgcccgctgctggacaggggctcggctgttgggcactgacaattccgtggtgttgtcggggaaatcatcgtcctttccttggctgctcgcctgtgttgccacctggattctgcgcgggacgtccttctgctacgtcccttcggccctcaatccagcggaccttccttcccgcggcctgctgccggctctgcggcctcttccgcgtcttcgccttcgccctcagacgagtcggatctccctttgggccgcctccccgcgtcgactttaagaccaatgacttacaaggcagctgtagatcttagccactttttaaaagaaaaggggggactggaagggctaattcactcccaacgaagacaagatttcaccattatcgtttcagacccacctcccaaccccgaggggacccagagagggcctatttcccatgattccttcatatttgcatatacgatacaaggctgttagagagataattagaattaatttgactgtaaacacaaagatattagtacaaaatacgtgacgtagaaagtaataatttcttgggtagtttgcagttttaaaattatgttttaaaatggactatcatatgcttaccgtaacttgaaagtatttcgatttcttggctttatatatcttgtggaaaggacgaaacaccggagacggttgtaaatgagcacacaaaatacacatgctaaaatattatattctatgacctttataaaatcaaccaaaatcttctttttaataactttagtatcaataattagaatttttatgttcctttttgcaaacttttaataaaaatgagcaaaataaaaaaacgctagttttagtaactcgcgttgttttcttcacctttaataatagctactccaccacttgttcctaagcggtcagctcctgcttcaatcattttttgagcatcttcaaatgttctaactccaccagctgctttaactaaagcattgtctttaacaactgacttcattagtttaacatcttcaaatgttgcacctgattttgaaaatcctgttgatgttttaacaaattctaatccagcttcaacagctatttcacaagctttcatgatttcttcttttgttaataaacaattttccataatacatttaacaacatgtgatccagctgctttttttacagctttcatgtcttctaaaactaattcataatttttgtcttttaatgcaccaatatttaataccatatcaatttctgttgcaccatctttaattgcttcagaaacttcgaatgcttttgtagctgttgtgcatgcacctagaggaaaacctacaacatttgttattcctacatttgtgccttttaataattctttacaatagcttgttcaatatgaattaacacaaactgttgcaaaatcaaattcaattgcttcatcacataattgtttaatttcagctttcgtagcatcttgttttaataatgtgtgatctatatatttgtttagtttcattttttctcctatatattcatttttaattttaattctttaataatttcgtctactttaactttagcgttttgaacagattcaccaacacctataaaataaatttttagtttaggttcagttccacttgggcgaacagcaaatcatgacttatcttctaaataaaattttagtaagtcttgtcctggcatattatacattccatcgatgtagtcttcaacattaacaactttaagtccagcaatttgagttaagggtgttgctctcaatgatttcattaatggttcaatttttaatttcttttcttctggtttaaaattcaagtttaaagtgaaagtgtaatatgcacccatttctttaaataaatcttctaaatagtctactaatgttttattttgttttttataaaatcaagcagcctctgctattaatatagaagcttgtattccatctttatctctagctgagtcatcaattacatatccataactttcttcataagcaaaaacaaaatttaatccgttatcttcttctttagcaatttctctacccattcatttaaatccagttaaagtttttacaatattaactccatatttttcatgagcgattctatcacccaaatcacttgttacaaaacttgaatatagagccggattttttggaatgctatttaagcgttttagatttgataattttcaatcaattaaaattggtcctgtttgatttccatctaatcttacaaaatgaccatcatgttttattgccattccaaatctgtcagcatctgggtcattcataataataatatctgcatcatgtttaataccatattcaagcggtatttttcatgcaggatcaaattctggatttggatttacaacatttttaaatgtttcatcttcaaatgcatgctcttcaacctcaataacgttatatcctgattcacgtaatatttttggggtaaatttagttcctgttccattaactgcgctaaaaataatttttaaatcttttttagcttcttgctcttttttgtacgtctctgtttaagagctatgctggaaacagcatagcaagtttaaataaggctagtccgttatcaacttgaaaaagtggcaccgagtcggtgcttttttaagcttggcgtaactagatcttgagacactgctttttgcttgtactgggtctctctggttagaccagatctgagcctgggagctctctggctaactagggaacccactgcttaagcctcaataaagcttgccttgagtgcttcaagtagtgtgtgcccgtctgttgtgtgactctggtaactagagatccctcagacccttttagtcagtgtggaaaatctctagcagtacgtatagtagttcatgtcatcttattattcagtatttataacttgcaaagaaatgaatatcagagagtgagaggaacttgtttattgcagcttataatggttacaaataaagcaatagcatcacaaatttcacaaataaagcatttttttcactgcattctagttgtggtttgtccaaactcatcaatgtatcttatcatgtctggctctagctatcccgcccctaactccgcccatcccgcccctaactccgcccagttccgcccattctccgccccatggctgactaattttttttatttatgcagaggccgaggccgcctcggcctctgagctattccagaagtagtgaggaggcttttttggaggcctagggacgtacccaattcgccctatagtgagtcgtattacgcgcgctcactggccgtcgttttacaacgtcgtgactgggaaaaccctggcgttacccaacttaatcgccttgcagcacatccccctttcgccagctggcgtaatagcgaagaggcccgcaccgatcgcccttcccaacagttgcgcagcctgaatggcgaatgggacgcgccctgtagcggcgcattaagcgcggcgggtgtggtggttacgcgcagcgtgaccgctacacttgccagcgccctagcgcccgctcctttcgctttcttcccttcctttctcgccacgttcgccggctttccccgtcaagctctaaatcgggggctccctttagggttccgatttagtgctttacggcacctcgaccccaaaaaacttgattagggtgatggttcacgtagtgggccatcgccctgatagacggtttttcgccctttgacgttggagtccacgttctttaatagtggactcttgttccaaactggaacaacactcaaccctatctcggtctattcttttgatttataagggattttgccgatttcggcctattggttaaaaaatgagctgatttaacaaaaatttaacgcgaattttaacaaaatattaacgcttacaatttaggtggcacttttcggggaaatgtgcgcggaacccctatttgtttatttttctaaatacattcaaatatgtatccgctcatgagacaataaccctgataaatgcttcaataatattgaaaaaggaagagtatgagtattcaacatttccgtgtcgcccttattcccttttttgcggcattttgccttcctgtttttgctcacccagaaacgctggtgaaagtaaaagatgctgaagatcagttgggtgcacgagtgggttacatcgaactggatctcaacagcggtaagatccttgagagttttcgccccgaagaacgttttccaatgatgagcacttttaaagttctgctatgtggcgcggtattatcccgtattgacgccgggcaagagcaactcggtcgccgcatacactattctcagaatgacttggttgagtactcaccagtcacagaaaagcatcttacggatggcatgacagtaagagaattatgcagtgctgccataaccatgagtgataacactgcggccaacttacttctgacaacgatcggaggaccgaaggagctaaccgcttttttgcacaacatgggggatcatgtaactcgccttgatcgttgggaaccggagctgaatgaagccataccaaacgacgagcgtgacaccacgatgcctgtagcaatggcaacaacgttgcgcaaactattaactggcgaactacttactctagcttcccggcaacaattaatagactggatggaggcggataaagttgcaggaccacttctgcgctcggcccttccggctggctggtttattgctgataaatctggagccggtgagcgtgggtctcgcggtatcattgcagcactggggccagatggtaagccctcccgtatcgtagttatctacacgacggggagtcaggcaactatggatgaacgaaatagacagatcgctgagataggtgcctcactgattaagcattggtaactgtcagaccaagtttactcatatatactttagattgatttaaaacttcatttttaatttaaaaggatctaggtgaagatcctttttgataatctcatgaccaaaatcccttaacgtgagttttcgttccactgagcgtcagaccccgtagaaaagatcaaaggatcttcttgagatcctttttttctgcgcgtaatctgctgcttgcaaacaaaaaaaccaccgctaccagcggtggtttgtttgccggatcaagagctaccaactctttttccgaaggtaactggcttcagcagagcgcagataccaaatactgttcttctagtgtagccgtagttaggccaccacttcaagaactctgtagcaccgcctacatacctcgctctgctaatcctgttaccagtggctgctgccagtggcgataagtcgtgtcttaccgggttggactcaagacgatagttaccggataaggcgcagcggtcgggctgaacggggggttcgtgcacacagcccagcttggagcgaacgacctacaccgaactgagatacctacagcgtgagctatgagaaagcgccacgcttcccgaagggagaaaggcggacaggtatccggtaagcggcagggtcggaacaggagagcgcacgagggagcttccagggggaaacgcctggtatctttatagtcctgtcgggtttcgccacctctgacttgagcgtcgatttttgtgatgctcgtcaggggggcggagcctatggaaaaacgccagcaacgcggcctttttacggttcctggccttttgctggccttttgctcacatgttctttcctgcgttatcccctgattctgtggataaccgtattaccgcctttgagtgagctgataccgctcgccgcagccgaacgaccgagcgcagcgagtcagtgagcgaggaagcggaagagcgcccaatacgcaaaccgcctctccccgcgcgttggccgattcattaatgcagctggcacgacaggtttcccgactggaaagcgggcagtgagcgcaacgcaattaatgtgagttagctcactcattaggcaccccaggctttacactttatgcttccggctcgtatgttgtgtggaattgtgagcggataacaatttcacacaggaaacagctatgaccatgattacgccaagcgcgcaattaaccctcactaaagggaacaaaagctggagctgcaagcttaatgtagtcttatgcaatactcttgtagtcttgcaacatggtaacgatgagttagcaacatgccttacaaggagagaaaaagcaccgtgcatgccgattggtggaagtaaggtggtacgatcgtgccttattaggaaggcaacagacgggtctgacatggattggacgaaccactgaattgccgcattgcagagatattgtatttaagtgcctagctcgatacataaacgggtctctctggttagaccagatctgagcctgggagctctctggctaactagggaacccactgcttaagcctcaataaagcttgccttgagtgcttcaagtagtgtgtgcccgtctgttgtgtgactctggtaactagagatccctcagacccttttagtcagtgtggaaaatctctagcagtggcgcccgaacagggacttgaaagcgaaagggaaaccagaggagctctctcgacgcaggactcggcttgctgaagcgcgcacggcaagaggcgaggggcggcgactggtgagtacgccaaaaattttgactagcggaggctagaaggagagagatgggtgcgagagcgtcagtattaagcgggggagaattagatcgcgatgggaaaaaattcggttaaggccagggggaaagaaaaaatataaattaaaacatatagtatgggcaagcagggagctagaacgattcgcagttaatcctggcctgttagaaacatcagaaggctgtagacaaatactgggacagctacaaccatcccttcagacaggatcagaagaacttagatcattatataatacagtagcaaccctctattgtgtgcatcaaaggatagagataaaagacaccaaggaagctttagacaagatagaggaagagcaaaacaaaagtaagaccaccgcacagcaagcggccgctgatcttcagacctggaggaggagatatgagggacaattggagaagtgaattatataaatataaagtagtaaaaattgaaccattaggagtagcacccaccaaggcaaagagaagagtggtgcagagagaaaaaagagcagtgggaat
