# Supplementary figures and images for: Mislocalization of pathogenic RBM20 variants in dilated cardiomyopathy is caused by loss-of-interaction with Transportin-3
Source: Nat Commun. 2023 Jul 18;14:4312. doi: 10.1038/s41467-023-39965-6 (PMC10353998; doi:10.1038/s41467-023-39965-6)

Supplementary Figure 8b

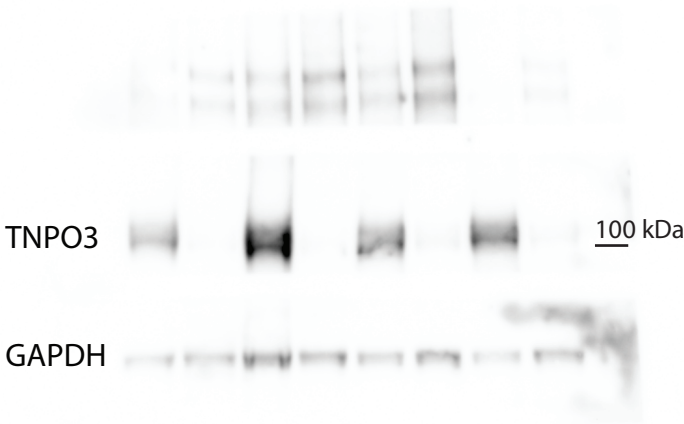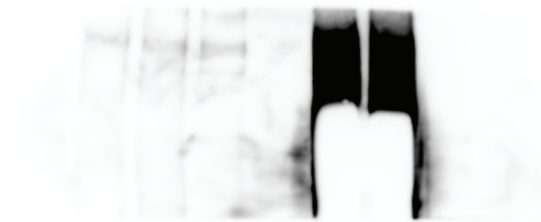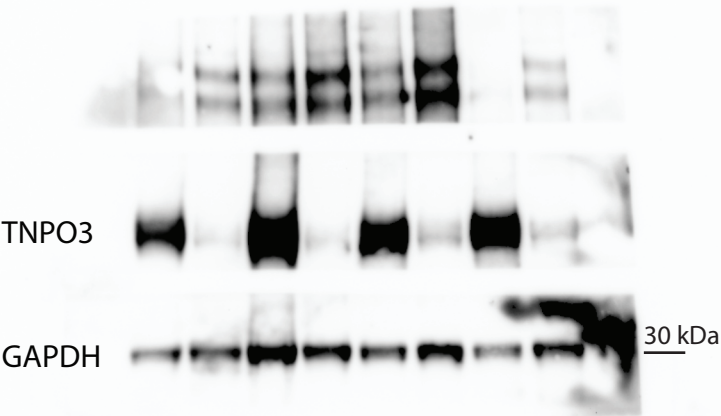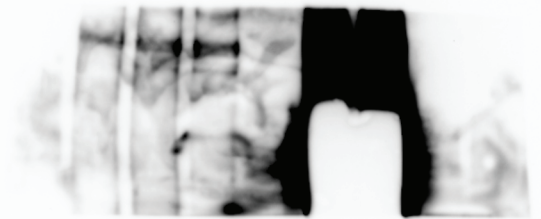

Supplement: Supplementary file 19 — Source data [file 41467_2023_39965_MOESM19_ESM.zip › Source_data/Supplementary Fig8b.pdf]

Supplementary Figure 8e

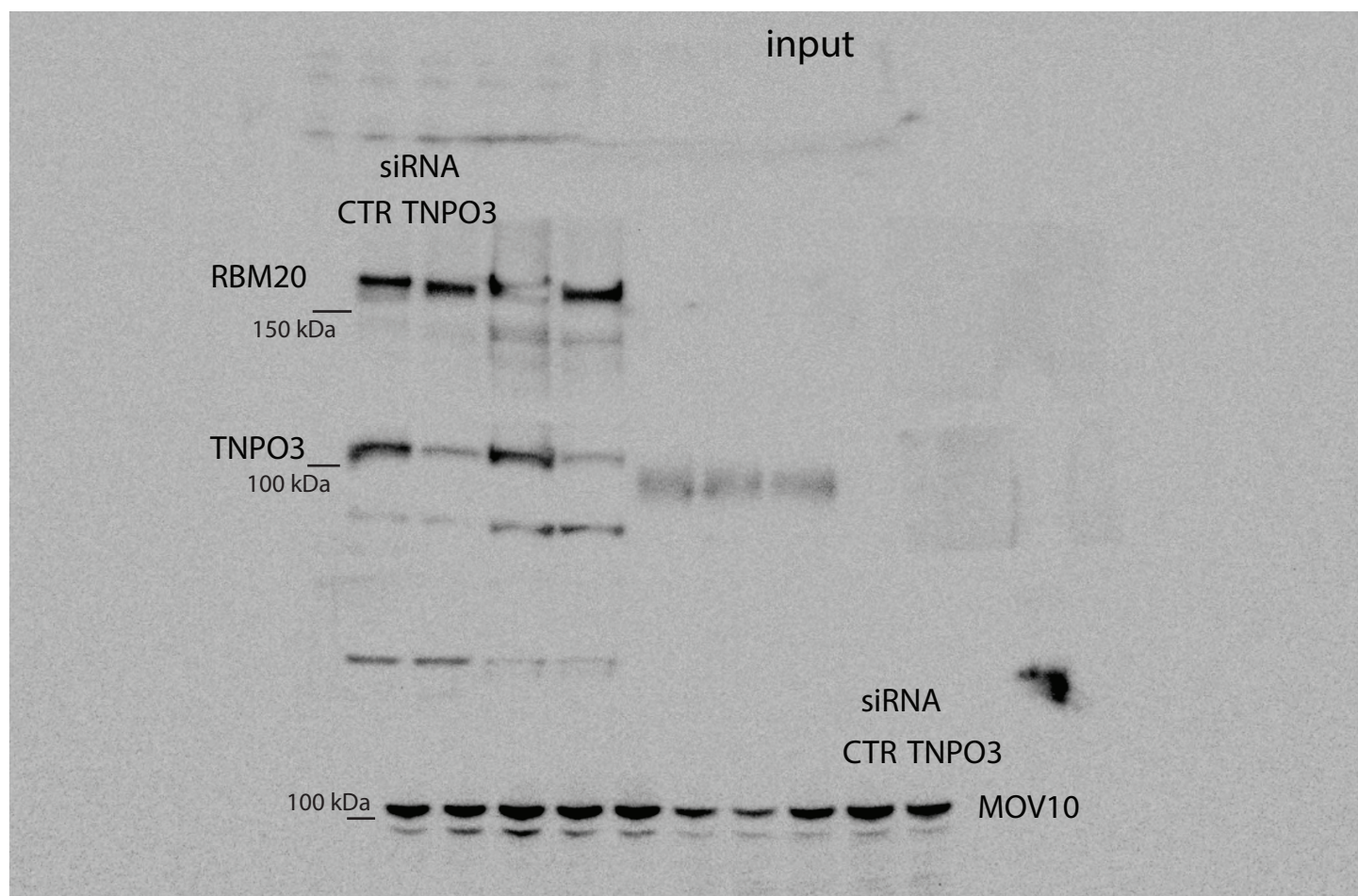

Supplementary Figure 8e

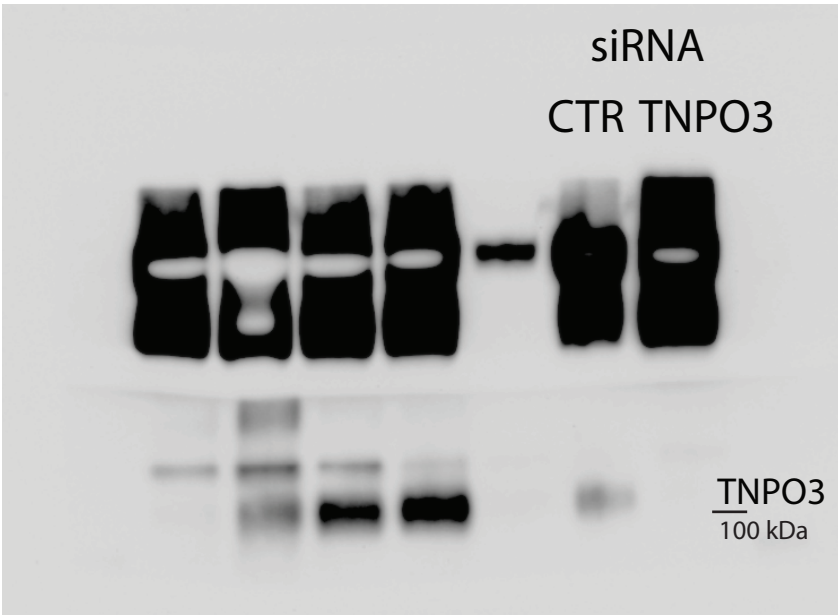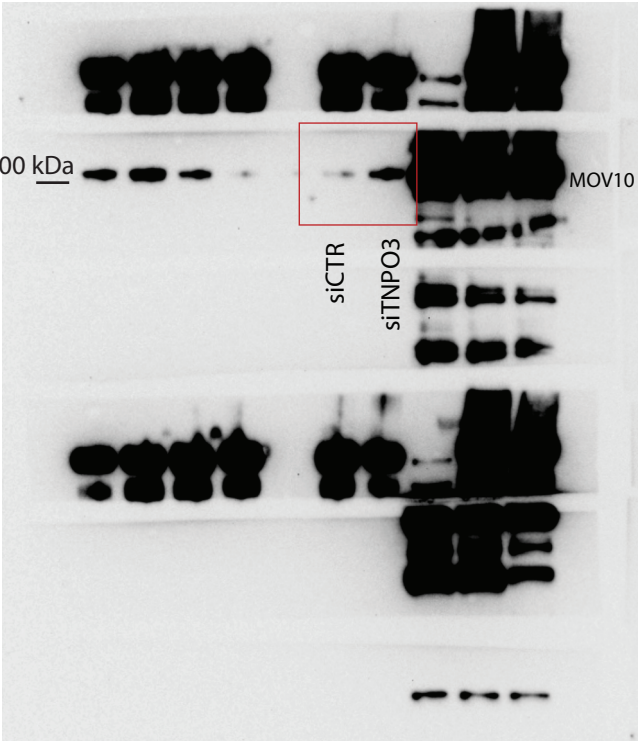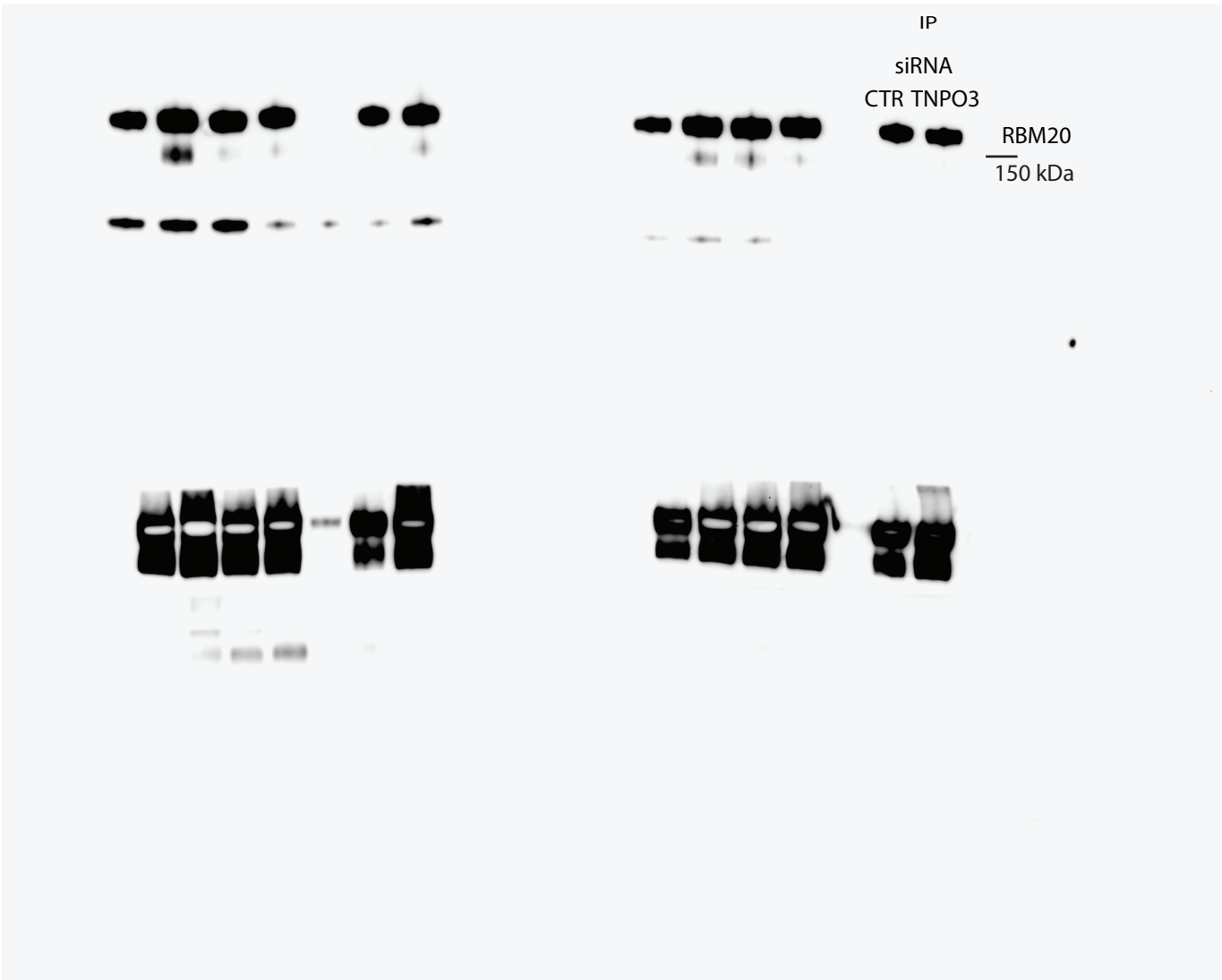

Supplement: Supplementary file 19 — Source data [file 41467_2023_39965_MOESM19_ESM.zip › Source_data/Supplementary Fig8e.pdf]

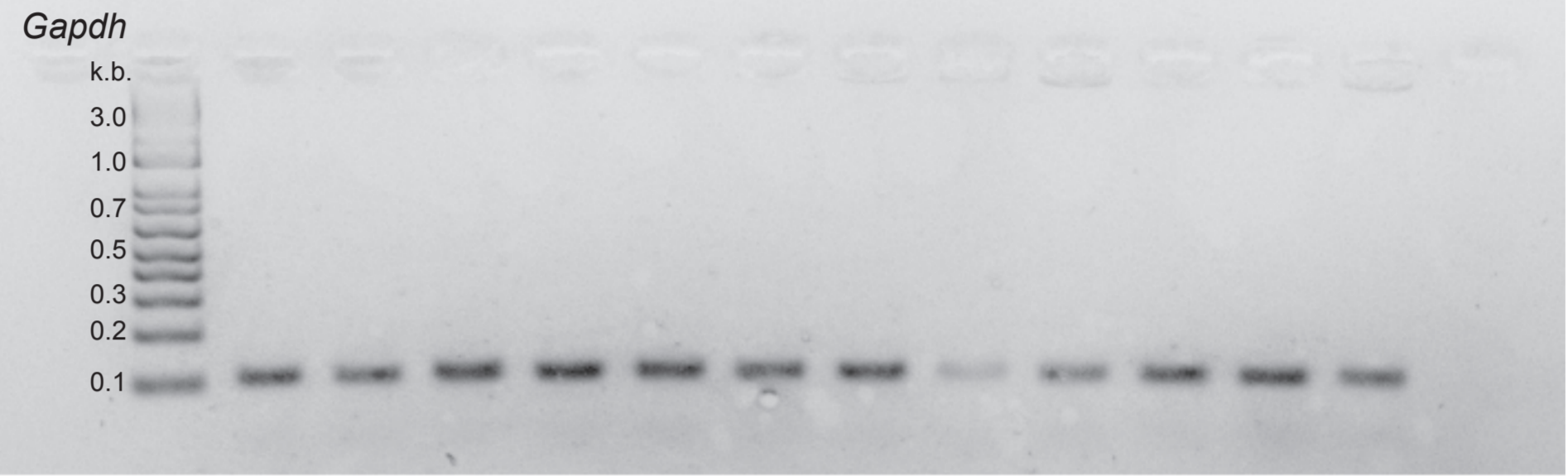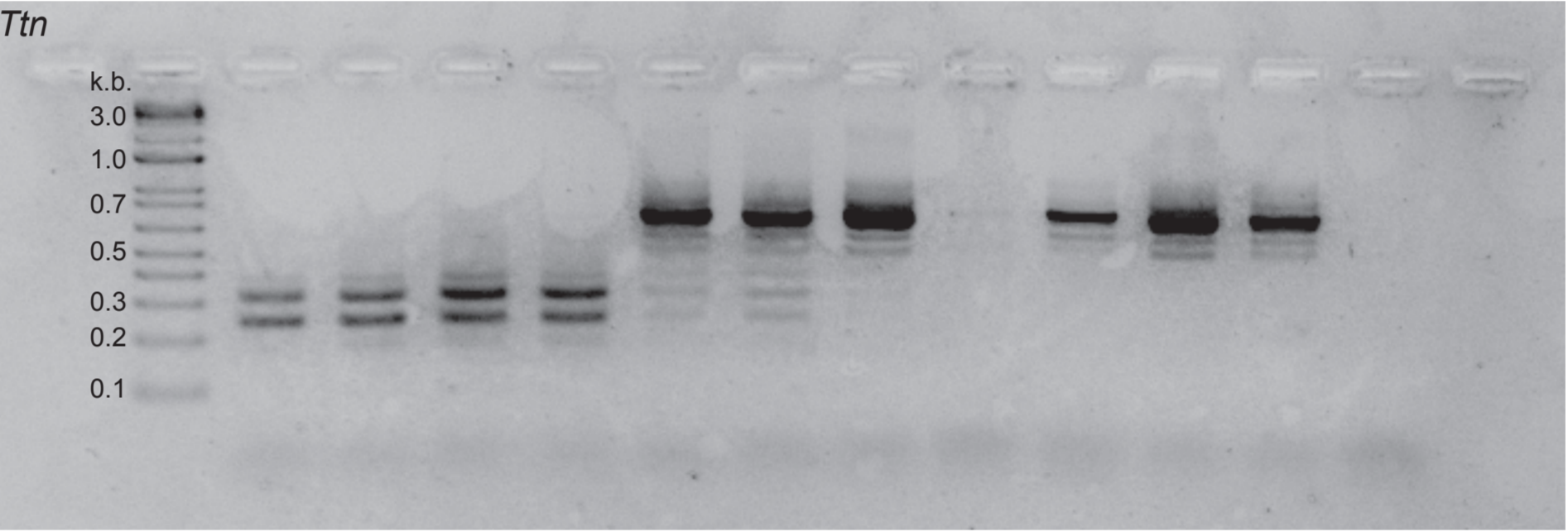

Supplement: Supplementary file 19 — Source data [file 41467_2023_39965_MOESM19_ESM.zip › Source_data/Fig5i.pdf]

Supplementary Figure 3a

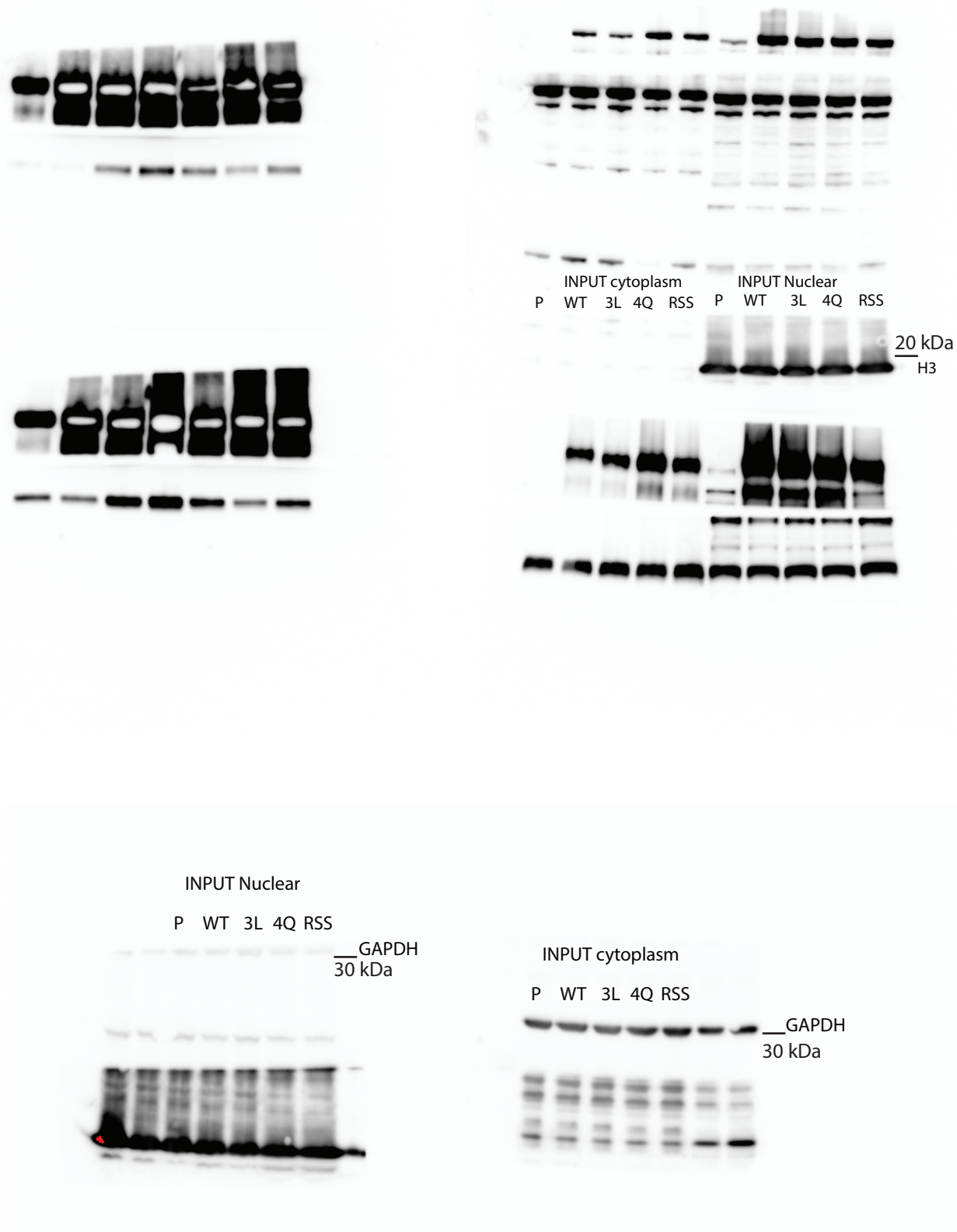

Supplementary Figure 3a

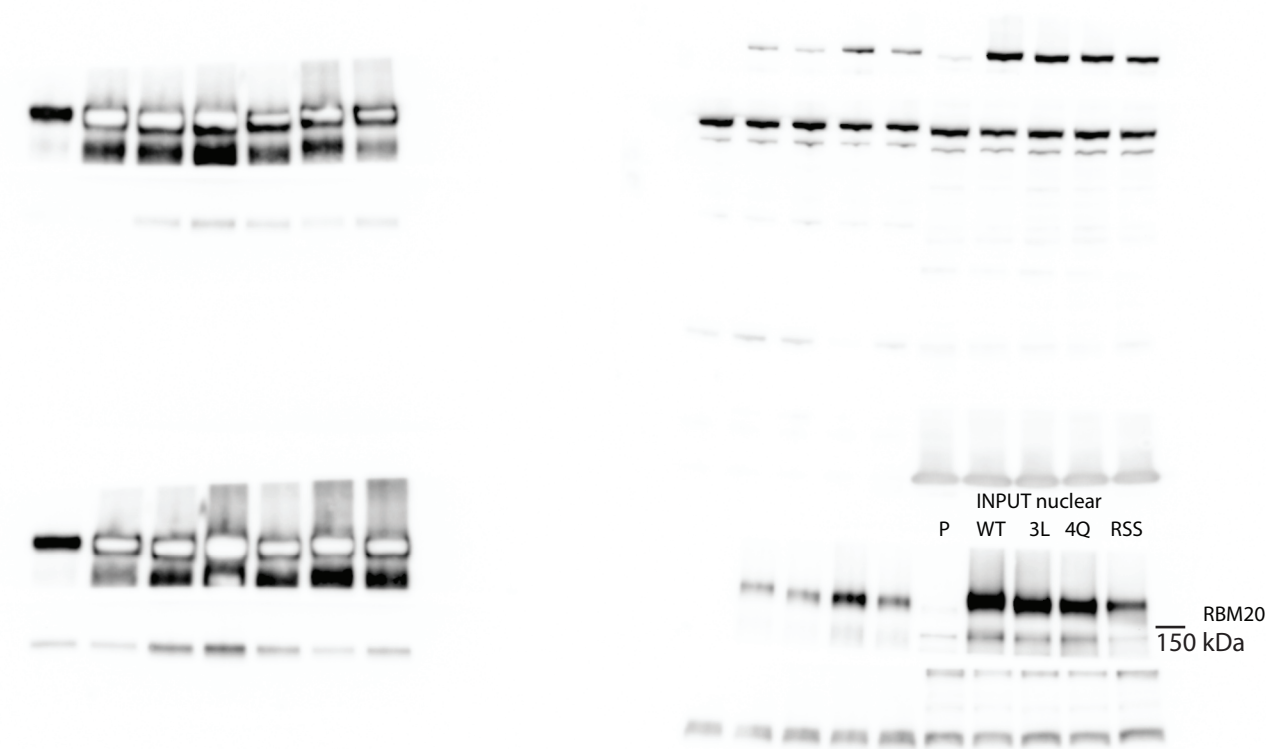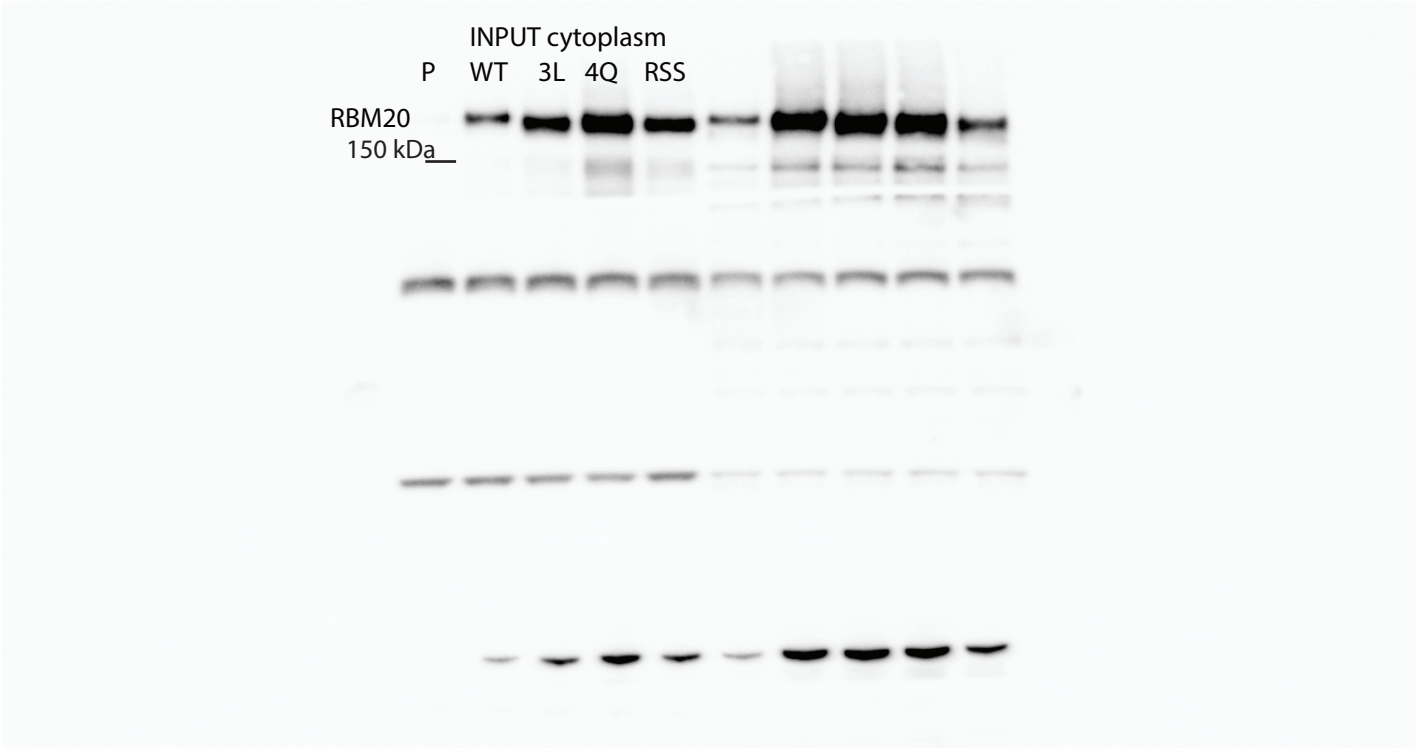

Supplement: Supplementary file 19 — Source data [file 41467_2023_39965_MOESM19_ESM.zip › Source_data/Supplementary Fig3a.pdf]

Figure 4j

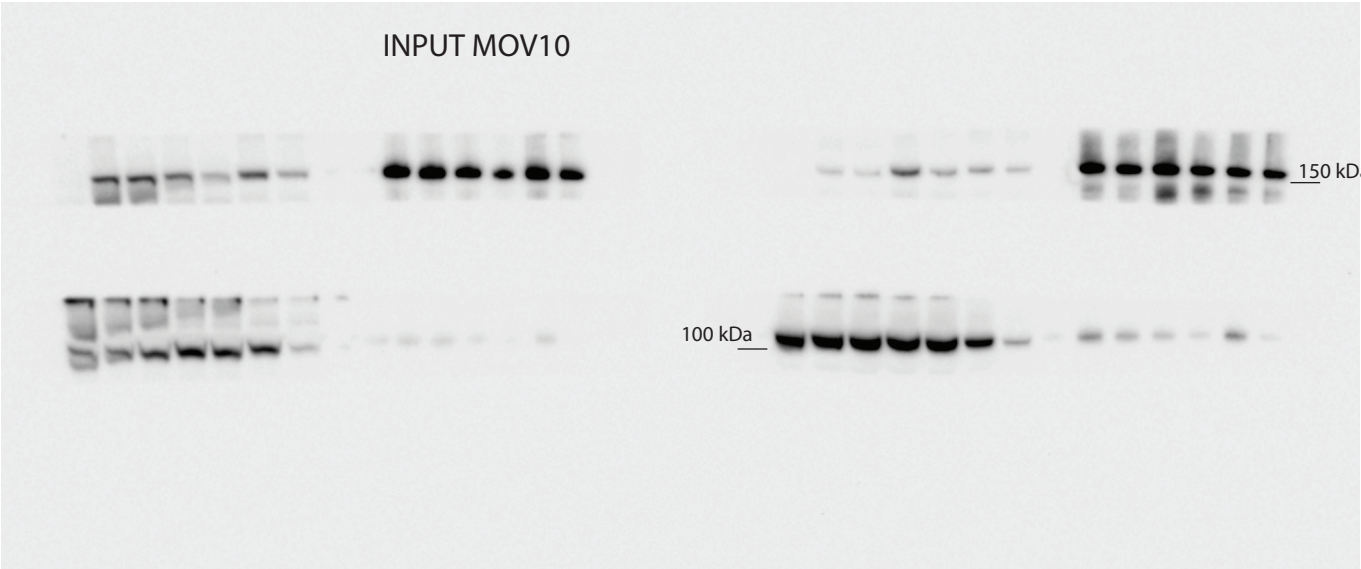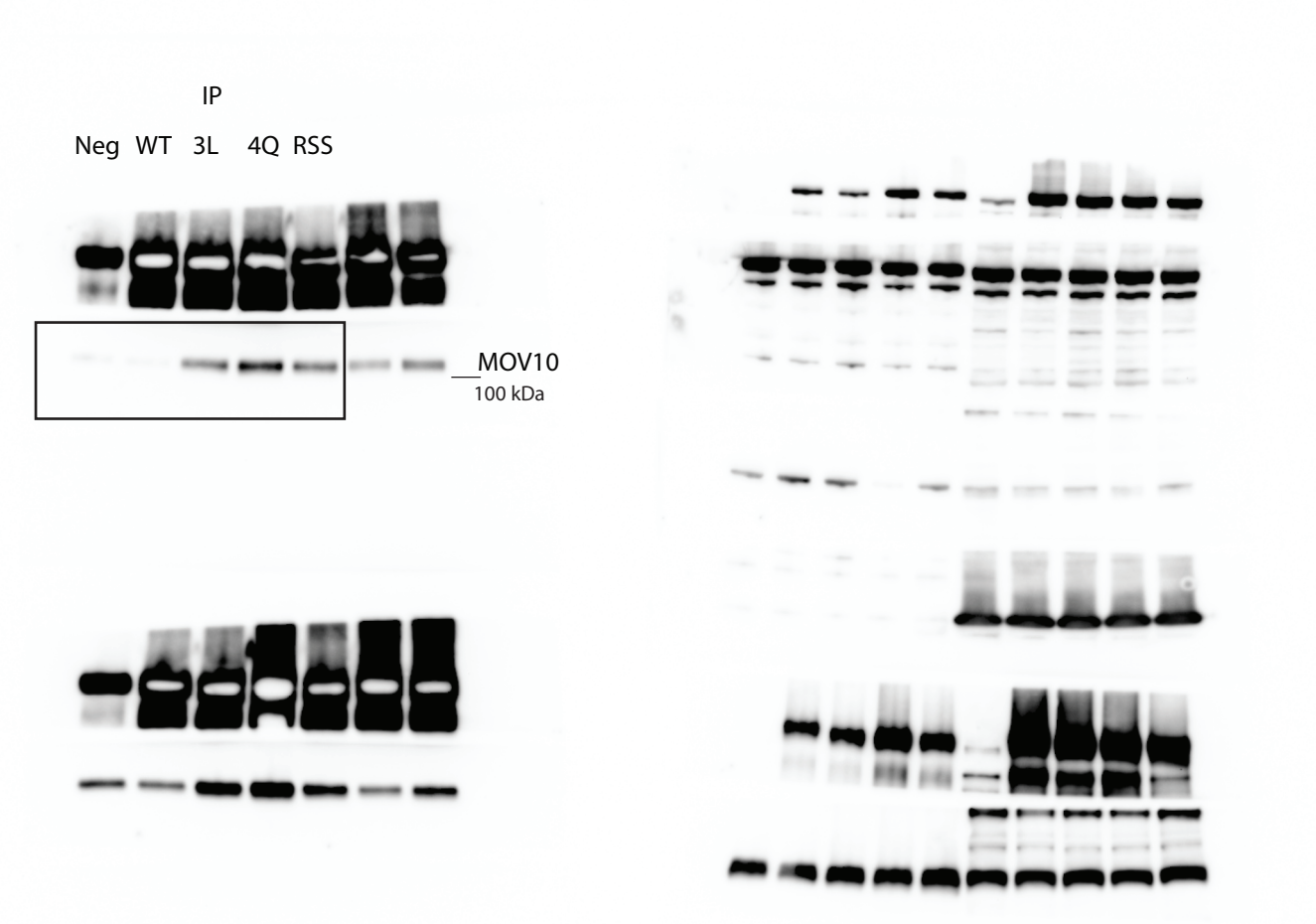

Figure 4j

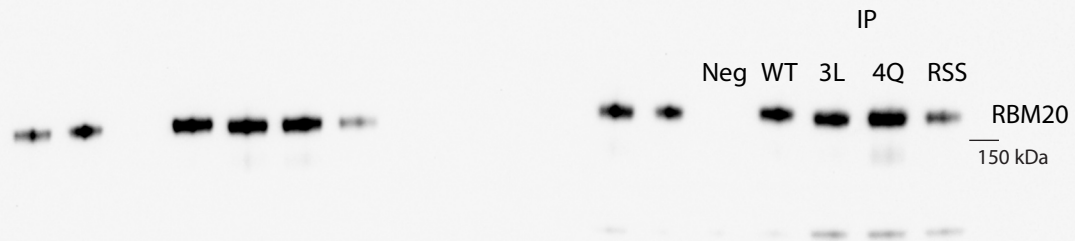

Figure 4j

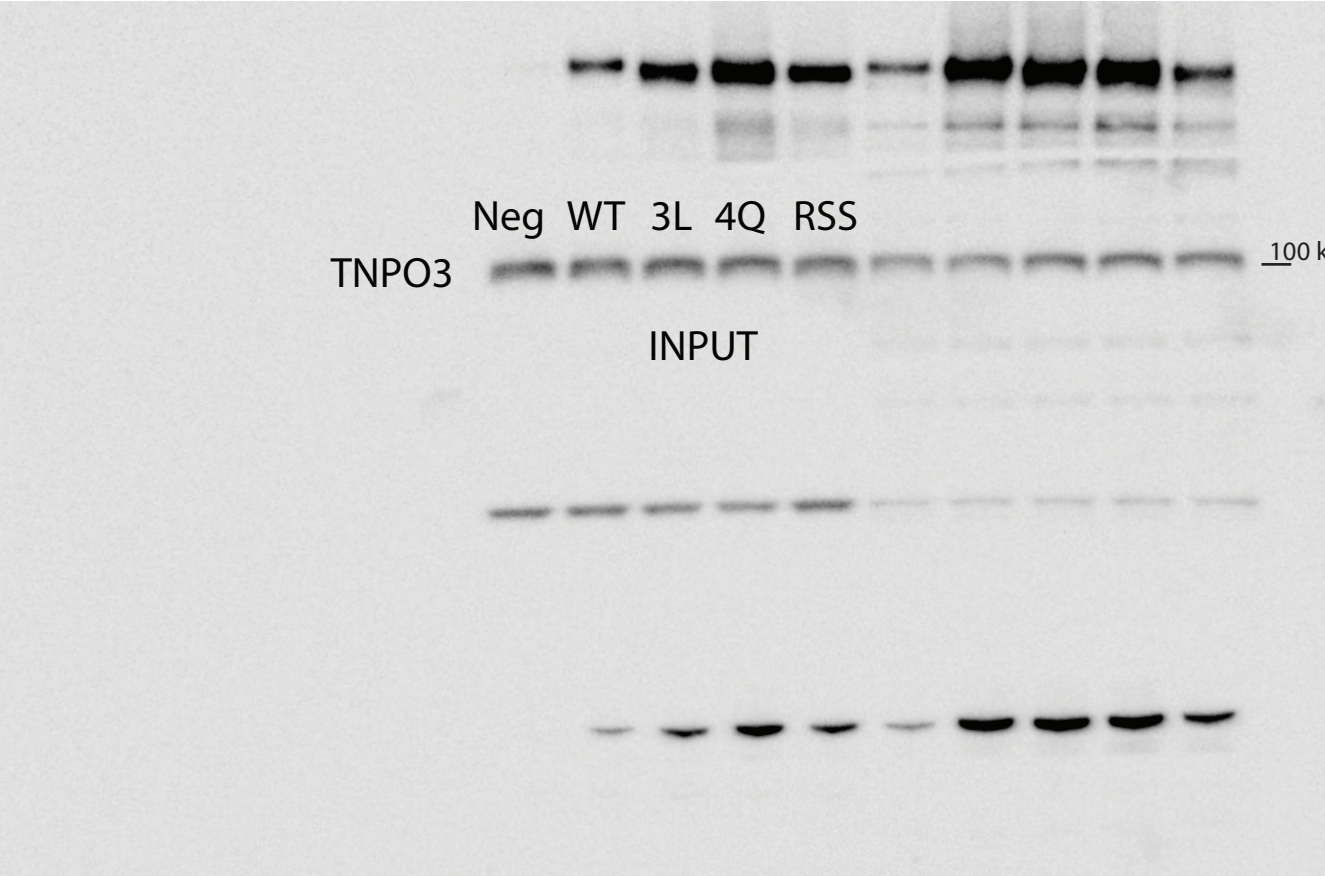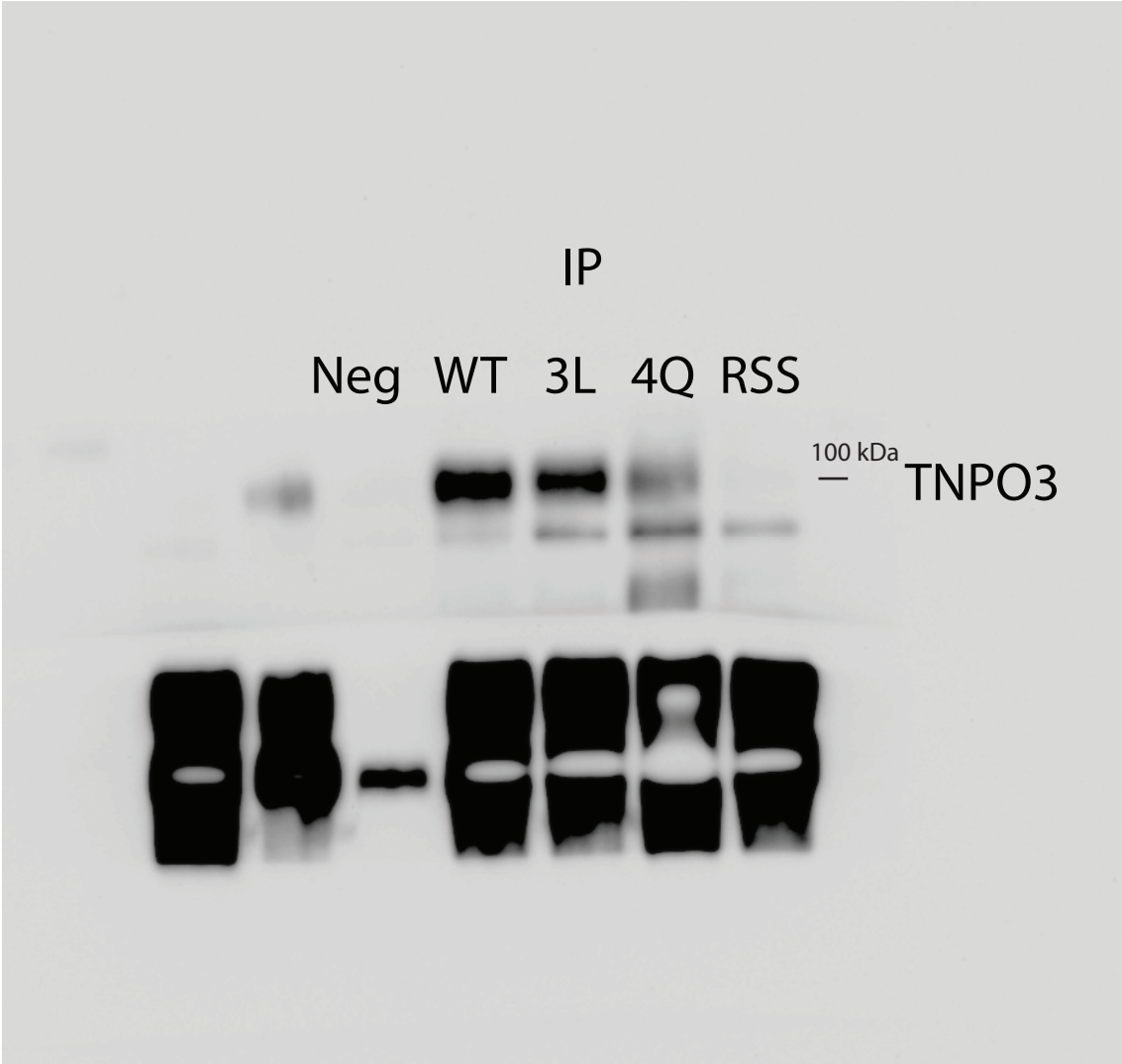

Supplement: Supplementary file 19 — Source data [file 41467_2023_39965_MOESM19_ESM.zip › Source_data/Fig4j.pdf]
